# ^1^H-NMR metabolomics analysis identifies hypoxanthine as a novel metastasis-associated metabolite in breast cancer

**DOI:** 10.1038/s41598-023-50866-y

**Published:** 2024-01-02

**Authors:** Sarra B. Shakartalla, Naglaa S. Ashmawy, Mohammad H. Semreen, Bahgat Fayed, Zainab M. Al Shareef, Manju N. Jayakumar, Saleh Ibrahim, Mohamed Rahmani, Rania Hamdy, Sameh S. M. Soliman

**Affiliations:** 1 Research Institute for Medical and Health Sciences, University of Sharjah, P.O. Box 27272, Sharjah, United Arab Emirates; 2 College of Medicine, University of Sharjah, P.O. Box 27272, Sharjah, United Arab Emirates; 3 Faculty of Pharmacy, University of Gezira, P.O. Box. 21111, Wadmedani, Sudan; 4 Department of Pharmaceutical Sciences, College of Pharmacy, Gulf Medical University, P.O. Box 4184, Ajman, United Arab Emirates; 5 Department of Pharmacognosy, Faculty of Pharmacy, Ain Shams University, Abbassia, P.O. Box 11566, Cairo, Egypt; 6 Chemistry of Natural and Microbial Product Department, National Research Centre, P.O. Box 12622, Cairo, Egypt; 7 Center for Biotechnology, Khalifa University, Abu Dhabi, United Arab Emirates; 8 College of Medicine and Health Sciences, Khalifa University, P.O. Box 127788, Abu Dhabi, United Arab Emirates; 9 Faculty of Pharmacy, Zagazig University, P.O. Box 44519, Zagazig, Egypt; 10 Department of Medicinal Chemistry, College of Pharmacy, University of Sharjah, P.O. Box 27272, Sharjah, United Arab Emirates

**Keywords:** Metabolomics, Breast cancer, Cancer microenvironment, Metastasis, Tumour biomarkers

## Abstract

Breast cancer is one of the leading causes of death in females, mainly because of metastasis. Oncometabolites, produced via metabolic reprogramming, can influence metastatic signaling cascades. Accordingly, and based on our previous results, we propose that metabolites from highly metastatic breast cancer cells behave differently from less-metastatic cells and may play a significant role in metastasis. For instance, we aim to identify these metabolites and their role in breast cancer metastasis. Less metastatic cells (MCF-7) were treated with metabolites secreted from highly metastatic cells (MDA-MB-231) and the gene expression of three epithelial-to-mesenchymal transition (EMT) markers including E-cadherin, N-cadherin and vimentin were examined. Some metabolites secreted from MDA-MB-231 cells significantly induced EMT activity. Specifically, hypoxanthine demonstrated a significant EMT effect and increased the migration and invasion effects of MCF-7 cells through a hypoxia-associated mechanism. Hypoxanthine exhibited pro-angiogenic effects via increasing the VEGF and PDGF gene expression and affected lipid metabolism by increasing the gene expression of PCSK-9. Notably, knockdown of purine nucleoside phosphorylase, a gene encoding for an important enzyme in the biosynthesis of hypoxanthine, and inhibition of hypoxanthine uptake caused a significant decrease in hypoxanthine-associated EMT effects. Collectively for the first time, hypoxanthine was identified as a novel metastasis-associated metabolite in breast cancer cells and represents a promising target for diagnosis and therapy.

## Introduction

In 2020, breast cancer was identified as the most common cause of cancer-related death in females, comprising 15.5%^1^. Metastasis, the dissemination of cancer cells from primary tumor to distant sites, constitutes the majority of cancer related death (~ 90%), yet this complex process remains poorly understood^2^. Generally, the current treatments for metastatic cancer are not curative, they only slow its growth or spread. Metastasis is a multistep process including invasion, intravasation and extravasation^3^. The escape and detachment of cancer cells from the primary tumor resemble epithelial-mesenchymal transition (EMT) in which the epithelial cells acquire the morphology and gene expression of mesenchymal cells enabling them to migrate and invade the surrounding tissues^4^.

The metabolism in cancer cells is reprogramed to support their vast proliferation, making metabolic changes are key biomarkers in cancer progression^5^. Metabolic genotypes and phenotypes, in addition to proliferation and metastasis capabilities differ among breast cancer subtypes. Several studies provide ample evidence indicating that there is a complex interplay between cancer metastasis and metabolic reprogramming in cancer^6^. Metabolic reprogramming could drive metastasis by creating oncometabolites to manipulate metastatic signaling cascades through regulating gene expression^6^. Metabolites were reported to induce EMT in order to facilitate tumor invasion^6^. 2- Hydroxyglutarate promotes EMT in colorectal cancer cells through epigenetic activation of zinc-finger E-box binding homeobox (ZEB) transcription factor^7^. Fumarate and succinate were also reported to promote EMT in colorectal cancer and renal cancer cells, respectively, via epigenetic modification and activation of EMT-associated signaling pathways^8,9^. Ana and her colleagues identified that the accumulation of methyl malonate is age-associated, which facilitates tumor invasion and metastasis^10^. This is initiated by the ability of methyl malonate to induce EMT via increasing SRY-box transcription factor 4 (SOX4) expression^10^.

Previously, we have also reported that metabolites secreted from MCF-7 cells are different from those secreted from normal cells^11^. Extracellular metabolites of MCF-7 cells reveal improved anticancer activity and lower toxicity on normal cells^11^. Therefore, the signaling contribution of breast cancer cell metabolites in metastasis requires further investigation. In this study, we hypothesize that metabolites from breast cancer cells with high metastatic potential behave differently from less-metastatic breast cancer cells and may play a significant role in metastasis.

## Results

**Figure 1:**
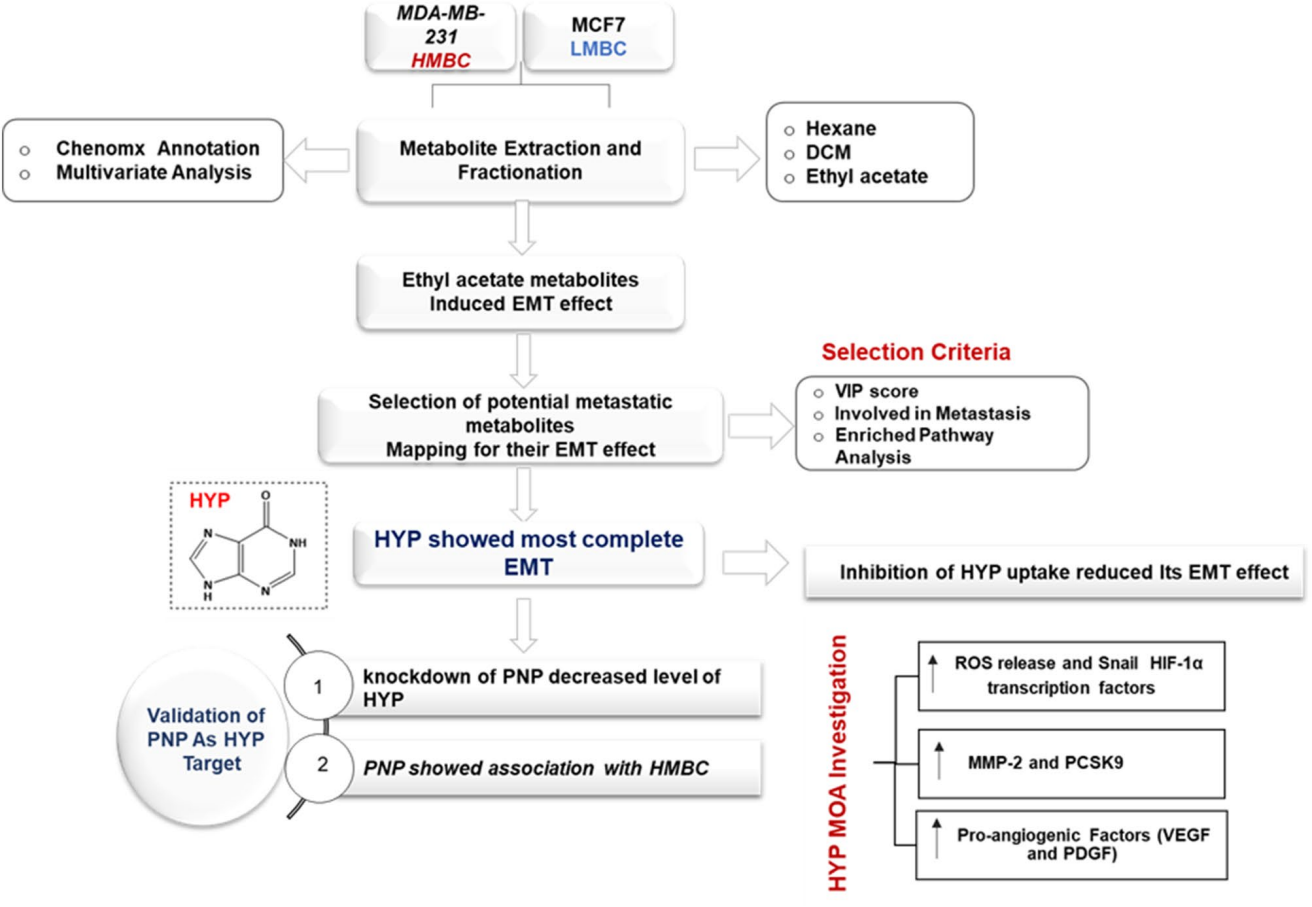
Schematic representation of the study workflow. HMBC; high-metastatic breast cancer cells and LMBC; low metastatic breast cancer cells.

The study workflow is illustrated in Fig. 1.

### Metabolites in the ethyl acetate fraction of MDA-MB-231 conditioned media induced EMT effect

**Figure 2:**
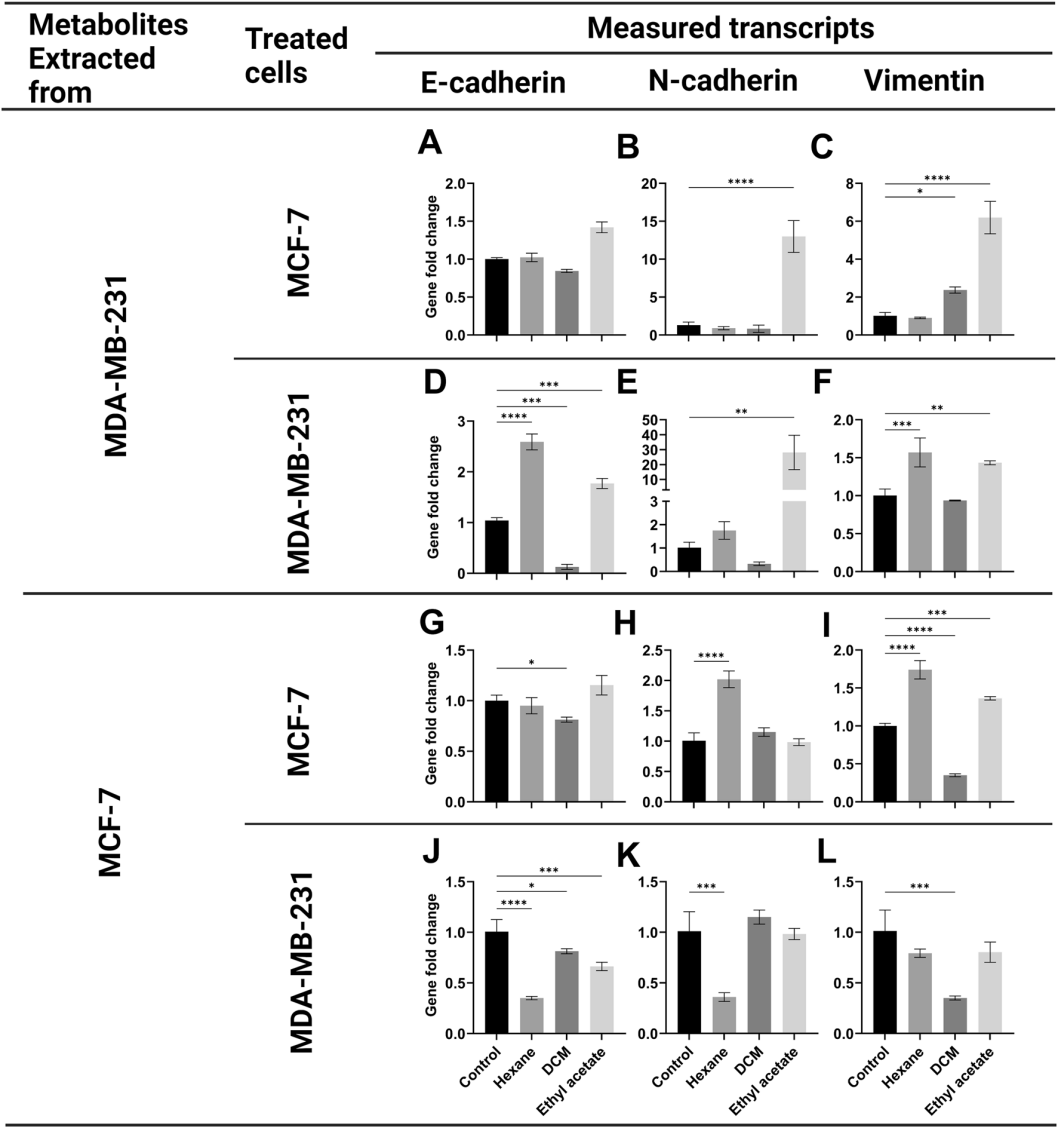
Gene expression analysis of E-cadherin, N-cadherin, and vimentin using qPCR for MCF-7 or MDA-MB-231 cells treated with the extracellular metabolites released from (**A**–**F**) MDA-MB-231 cells or (**G**–**L**) MCF-7 cells and extracted using hexane, DCM, or ethyl acetate compared to untreated cells (control). Metabolites extracted refer to the cell type from which the metabolites were extracted. Treated cells refer to the type of cells that are treated with metabolite extracts. All treatments were performed in triplicates. Changes in the gene expression is expressed as gene fold change. Data were presented as mean ± SEM (n = 3). The analysis was performed using one-way analysis of variance (ANOVA) and Tukey’s multiple comparison test. *P* value ≤ 0.05 was considered significant. * Reveals that *P* value < 0.05, ** reveals that *P* value < 0.01, *** reveals that *P* value < 0.001, **** reveals that *P* value < 0.0001.

Comparing the effect of different extracts from highly metastatic (MDA-MB-231) conditioned media versus those extracted from less metastatic (MCF-7) conditioned media, one can conclude that metabolites of ethyl acetate extract of MDA-MB-231 conditioned media caused significant hybrid EMT effect on both cell lines (Fig. 2). These results encouraged the investigation to identify the metastasis-associated metabolite(s).

### Extracellular metabolites released from MDA-MB-231 cells are significantly different than those from MCF-7 cells

**Figure 3:**
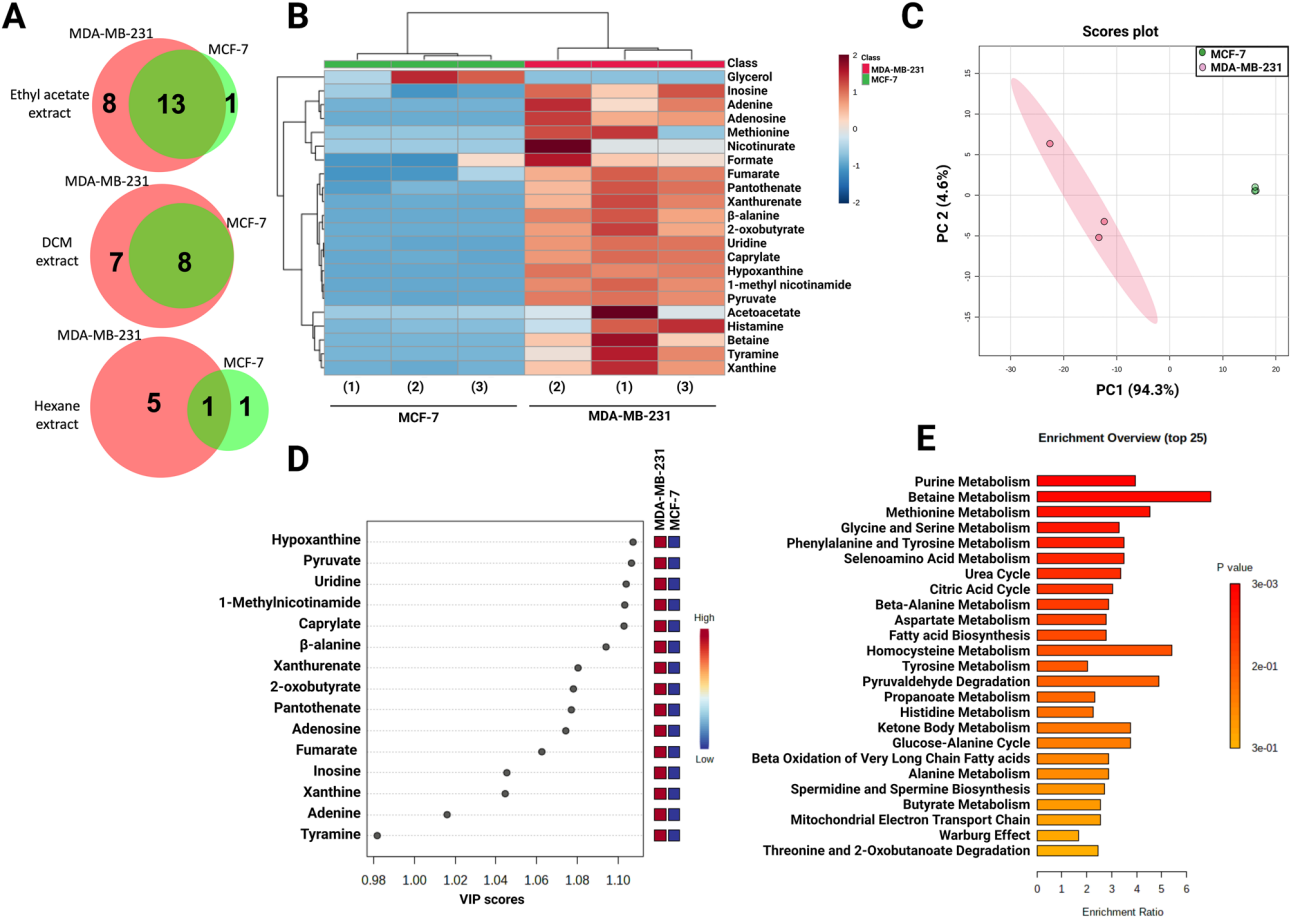
Metabolite distribution in MCF-7 and MDA-MB-231 conditioned media. (**A**) Venn diagram showing the number of metabolites in ethyl acetate, DCM, and hexane fractions in MCF-7 and MDA-MB-231 conditioned media. (**B**) Unsupervised hierarchical clustering and heatmap analysis of extracellular metabolites released from MDA-MB-231 and MCF-7 cells extracted using ethyl acetate fractions. The values of metabolites represent the average concentration (mM) in MCF-7 or MDA-MB-231 conditioned media. Different colors represent the concentration of different metabolites, where red represents a high concentration and blue represents a low concentration. (**C**) PCA of the metabolite profiling of MCF-7 and MDA-MB-231 conditioned media extracted using ethyl acetate. The red color indicates MDA-MB-231, and green indicates MCF-7 conditioned media. (**D**) VIP score plot of MCF-7 and MDA-MB-231 conditioned media metabolites. (**E**) Metabolite set enrichment analysis (MSEA) ranked by Holm* P* value showed the top 25 enriched pathways by ethyl acetate fraction of MDA-MB-231 conditioned media.

Comparative metabolomics analysis was carried out between the less metastatic MCF-7 and the highly metastatic MDA-MB-231 conditioned media using ^1^H-NMR (Figs. S1 and S2). The number and name of metabolites in each extract of MCF-7 and MDA-MB-231 conditioned media are shown in Fig. 3A and Table S2. Several metabolites were exclusively identified in MDA-MB-231 conditioned media with metastatic potential. These metabolites included 1-methynicotinamide, adenine, adenosine, betaine, hypoxanthine, xanthine, methionine, nicotinurate, and tyramine. However, glycerol was only detected in MCF-7 conditioned media.

Because of the significant effect of ethyl acetate metabolites extracts and their variable metabolite levels and numbers, metabolites from ethyl acetate extract were deeply investigated as below.

### Multivariate analysis confirmed the significant difference between the ethyl acetate metabolites released from the highly metastatic MDA-MB-231 compared to the less metastatic MCF-7 cells

Multivariate analyses namely hierarchical cluster analysis (HCA), partial least squares-discriminant analysis (PLS-DA), and principal component analysis (PCA) were conducted to investigate the overall difference in the metabolites of the ethyl acetate extracts between the highly metastatic MDA-MB-231 and the less metastatic MCF-7 conditioned media. HCA classified the metabolites into two main clusters; the first cluster represented a group of metabolites that have their highest abundance in MDA-MB-231 and the second cluster included the metabolites that were detected in MCF-7 conditioned media (heatmap in Fig. 3B). PCA was used to identify inter and intragroup variation. The primary component showed 94.3% variation, while the second component showed 4.6% variation (Fig. 3C). Supervised multivariate analysis, PLS-DA, was subsequently used to select the most specific metabolites from the ethyl acetate fractions that differentiate MDA-MB-231 from MCF-7 conditioned media. The scores plot showed 82.6% of the variance (Fig. S3). VIP was chosen as criteria for selecting the most important variables of the PLS-DA model (Fig. 3D). The most significantly different metabolites with VIP score more than 1 were hypoxanthine, xanthine, pyruvate, uridine, 1-methylnicotinamide, caprylate, β-alanine, xanthurenate, 2-oxobutyrate, pantothenate, adenosine, fumarate, inosine, xanthine, and adenine.

### Metabolic pathways and functional enrichment analysis supported the metastatic activity of metabolites from the ethyl acetate extract of MDA-MB-231 conditioned media

Metabolic pathways and functional enrichment analyses were carried out to discover the pathways enriched by the extracellular metabolites released from MDA-MB-231 cells using Metaboanalyst (Fig. 3E). The top enriched pathways by metabolites in the ethyl acetate fraction of MDA-MB-231 conditioned media were purine metabolism, betaine metabolism, methionine metabolism, glycine, and serine metabolism, which are all one-carbon metabolic pathways (Fig. 3E). One carbon metabolism was previously reported to contribute to the epigenetic dysregulation and acquisition of metastatic traits^12^.

### Ethyl acetate metabolites secreted from MDA-MB-231 cells significantly induce EMT

To identify the metabolites in the ethyl acetate extract of MDA-MB-231 conditioned media responsible for the EMT effects, the potential metastatic activity of the identified metabolites from the ethyl acetate extract of MDA-MB-231 conditioned media were searched in the literature (Table S3). The metabolites with predicted responsive metastatic activity were purchased and their EMT effects were tested separately. The EMT effects due to these metabolites at different concentrations (1, 10 or 100 µM) were then tested on the less metastatic MCF-7 cells. The results obtained indicated that xanthurenate, pyruvate, β-alanine, inosine, and uridine exhibited hybrid EMT activity (Fig. S4), while hypoxanthine showed the most and complete EMT effect and may exhibit a pro-metastatic activity. Therefore, hypoxanthine was further tested and the intracellular and extracellular amounts in MCF-7 and MDA-MB-231 cells were measured to identify if the amount of hypoxanthine inside the cells is positively correlated to metastatic potential of breast cancer cells and to determine the reasonable concentrations that should be used for cell culture treatment. The extra- and intracellular hypoxanthine levels in the high metastatic MDA-MB-231 cells and the less metastatic MCF-7 cells were measured using ^1^H-NMR (Fig. S5) and validated by LC–MS/MS (Figs. S6–S8). ^1^H-NMR results showed that the intracellular level of hypoxanthine inside MDA-MB-231 cells (0.55 ± 0.04 µM/ 3 × 10^5^ cells) was ~ 3 times higher than in MCF-7 cells (0.21 ± 0.05 µM/ 3 × 10^5^ cells). The extracellular level of hypoxanthine from MDA-MB-231 cells was 1.065 ± 0.04 µM/ 3 × 10^5^ cells, while hypoxanthine was not detected in the conditioned media of MCF-7 cells. These results were further validated by LC–MS/MS before and after spiking with 2 µM standard hypoxanthine (Figs. S6–S8). The results from LC–MS/MS showed that the intracellular level of hypoxanthine inside MDA-MB-231 cells after spiking was 4.8 µM ± 0.03 µM/ 1 × 10^6^ cells, which is also ~ 3 times higher than in MCF-7 cells (1.7 µM ± 0.05 µM/ 1 × 10^6^ cells). The extracellular level of hypoxanthine in MDA-MB-231 cells in the spiked sample was 6.10 ± 0.04 µM/1 × 10^6^. These findings indicated that the level of hypoxanthine released from the highly metastatic MDA-MB-231 cells was higher than the less metastatic MCF-7 cells, suggesting a strong correlation between hypoxanthine and metastasis. Based on these results, we propose that the secretion of hypoxanthine by highly metastatic cells (MDA-MB-231) and not by the low metastatic cells (MCF-7) contributes to the metastatic behavior in the tumor microenvironment of MDA-MB-231 cells by making the neighboring cells are more susceptible to the effect of hypoxanthine through ENT2 and the induction of EMT effect. Therefore, we assume that the inhibition of ENT2 can result in reduction/ inhibition of hypoxanthine uptake by the neighboring cells and hence inhibition of EMT effect. Furthermore, we also selected 0.01, 0.1, and 1 µM for cell culture treatment.

**Figure 4:**
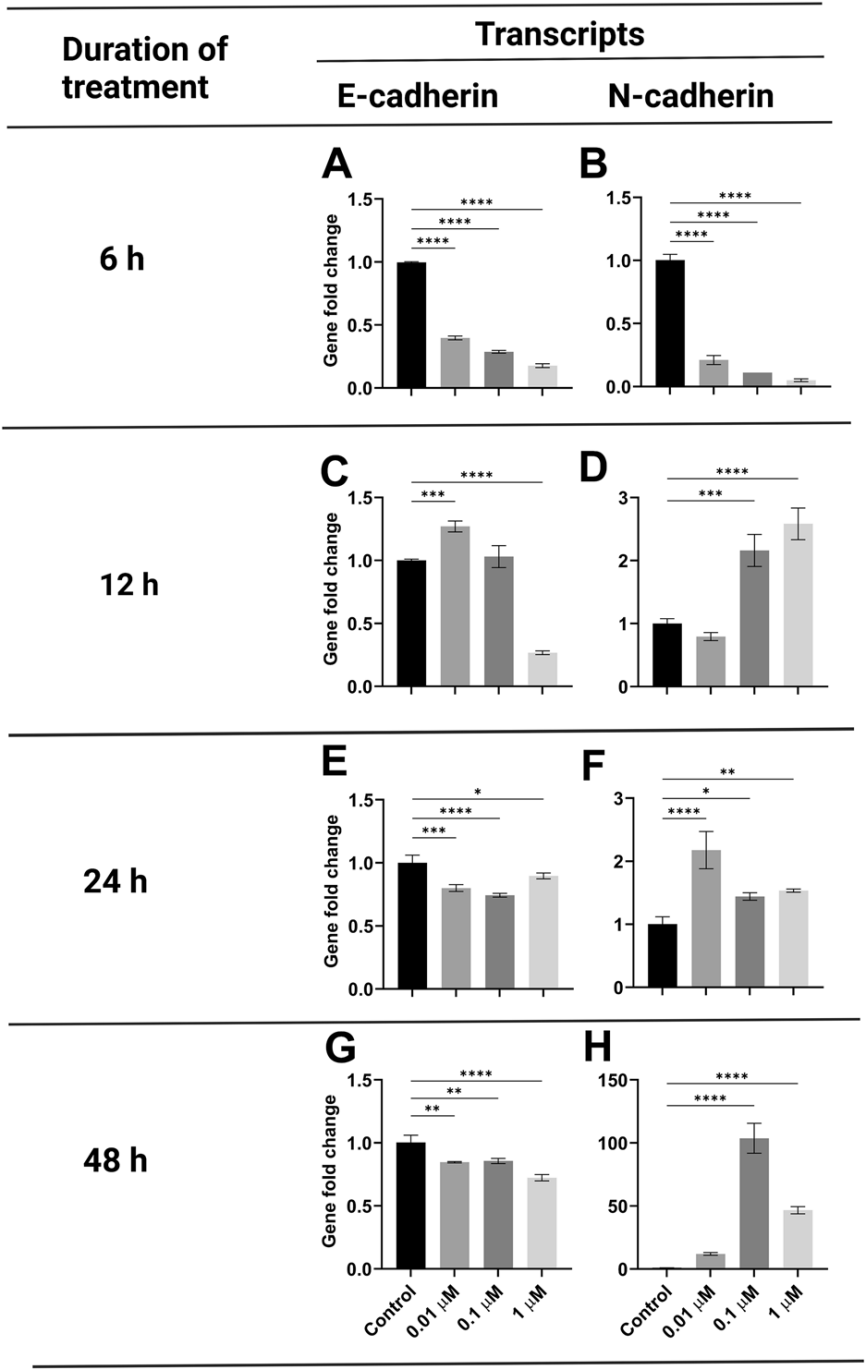
Gene expression analysis of E-cadherin and N-cadherin in MCF-7 treated with 0.01, 0.1 and 1 µM HYP at different incubation periods. The data were analyzed using one-way ANOVA. *P* value ≤ 0.05 was considered significant. * Reveals that *P* value < 0.05, ** reveals that *P* value < 0.01, *** reveals that *P* value < 0.001, **** reveals that *P* value < 0.0001.

Hypoxanthine was further tested at the selected concentrations (0.01, 0.1, and 1 µM) and different time points (6, 12, 24, and 48h) (Fig. 4A–H). More prominent EMT effects were demonstrated after 48h (Fig. 4G, H). Therefore, 48h duration time was selected in the downstream experiments.

### Hypoxanthine significantly induced EMT effects and enhanced the migration and invasion of MCF-7 cells

**Figure 5:**
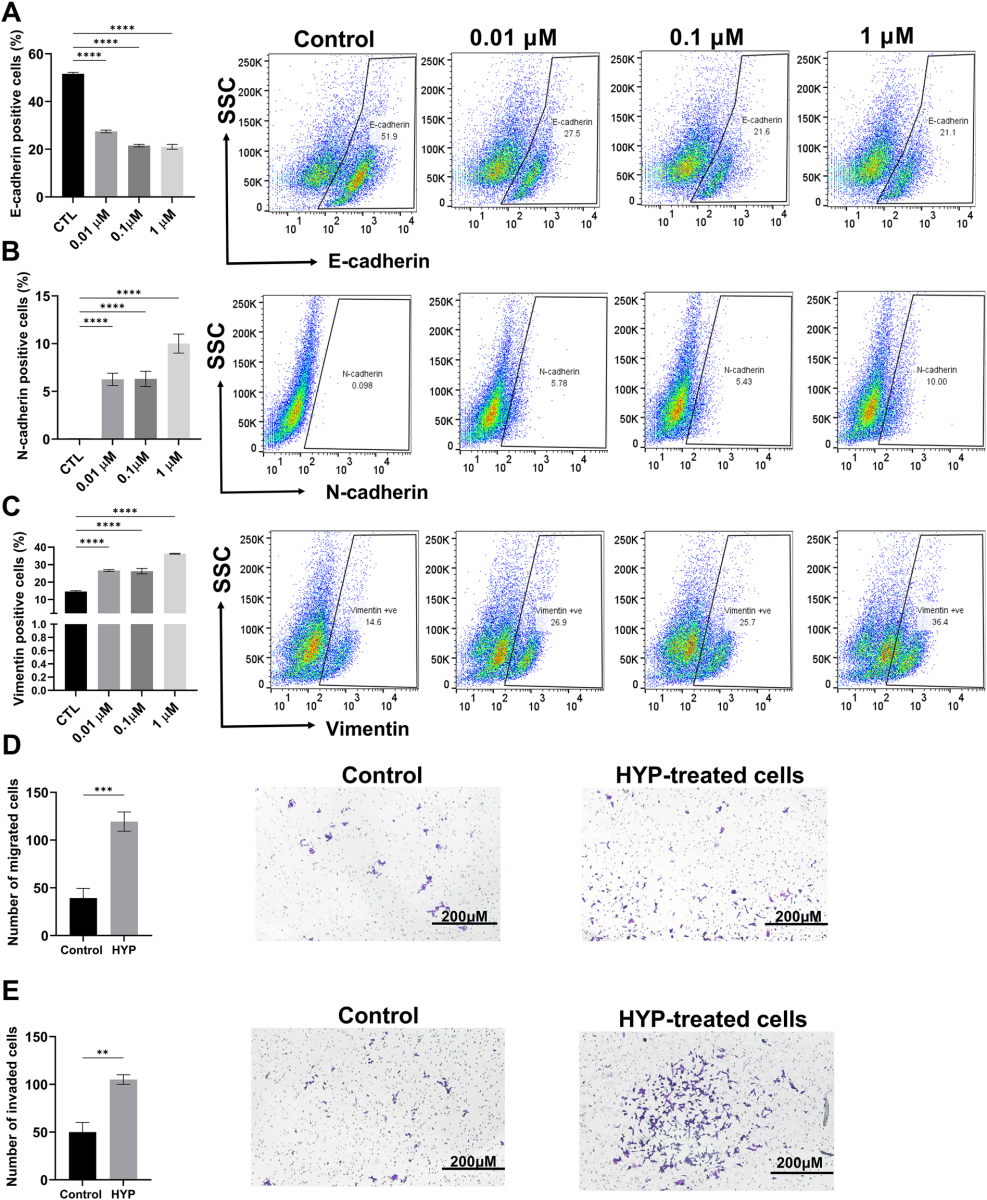
Hypoxanthine induces EMT, migration and invasion of MCF-7 cells. Flow cytometry analysis for testing the expression of (**A**) E-cadherin, (**B**) N-cadherin and (**C**) vimentin in MCF-7 cells treated with 0.01, 0.1 and 1 μM of hypoxanthine. (**D** and **E**) Transwell migration and invasion assay for MCF-7 cells treated with 0.1 μM of hypoxanthine. Data were presented as mean ± SEM (n = 3). Flow cytometry data were analyzed using one-way ANOVA and Tukey’s multiple comparison test and transwell invasion and migration assay was done using un-paired t-test. *P* value ≤ 0.05 was considered significant. * Reveals that *P* value < 0.05, ** reveals that *P* value < 0.01, *** reveals that *P* value < 0.001, **** reveals that *P* value < 0.0001.

To validate our results on the protein level, flow cytometry analysis of MCF-7 cells treated with different concentrations of hypoxanthine (0.01, 0.1, 1 µM) was conducted. Results showed significant (*P* < 0.0001) decrease in E-cadherin by ~ 0.4 folds (Fig. 5A), relative to vehicle treated cells and significant increase (*P* < 0.0001) in N-cadherin at 0.01, 0.1 µM by ~ 58 folds and at 1 µM by 102 folds (Fig. 5B). A significant increase (*P* < 0.0001) in the expression of vimentin at 0.01, 0.1, and 1 µM by 1.8, 1.76, 2.49% folds was observed, respectively (Fig. 5C). To identify the ability of hypoxanthine to promote migration and invasion of MCF-7 cells, transwell migration and Matrigel invasion assays were conducted. Hypoxanthine caused significant increase in the migration (*P* < 0.001) and invasion (*P* < 0.01) of MCF-7 cells by 80 and 55%, respectively (Fig. 5D, E).

### Inhibition of hypoxanthine uptake by MCF-7 cells reduced the hypoxanthine-induced EMT effect

**Figure 6:**
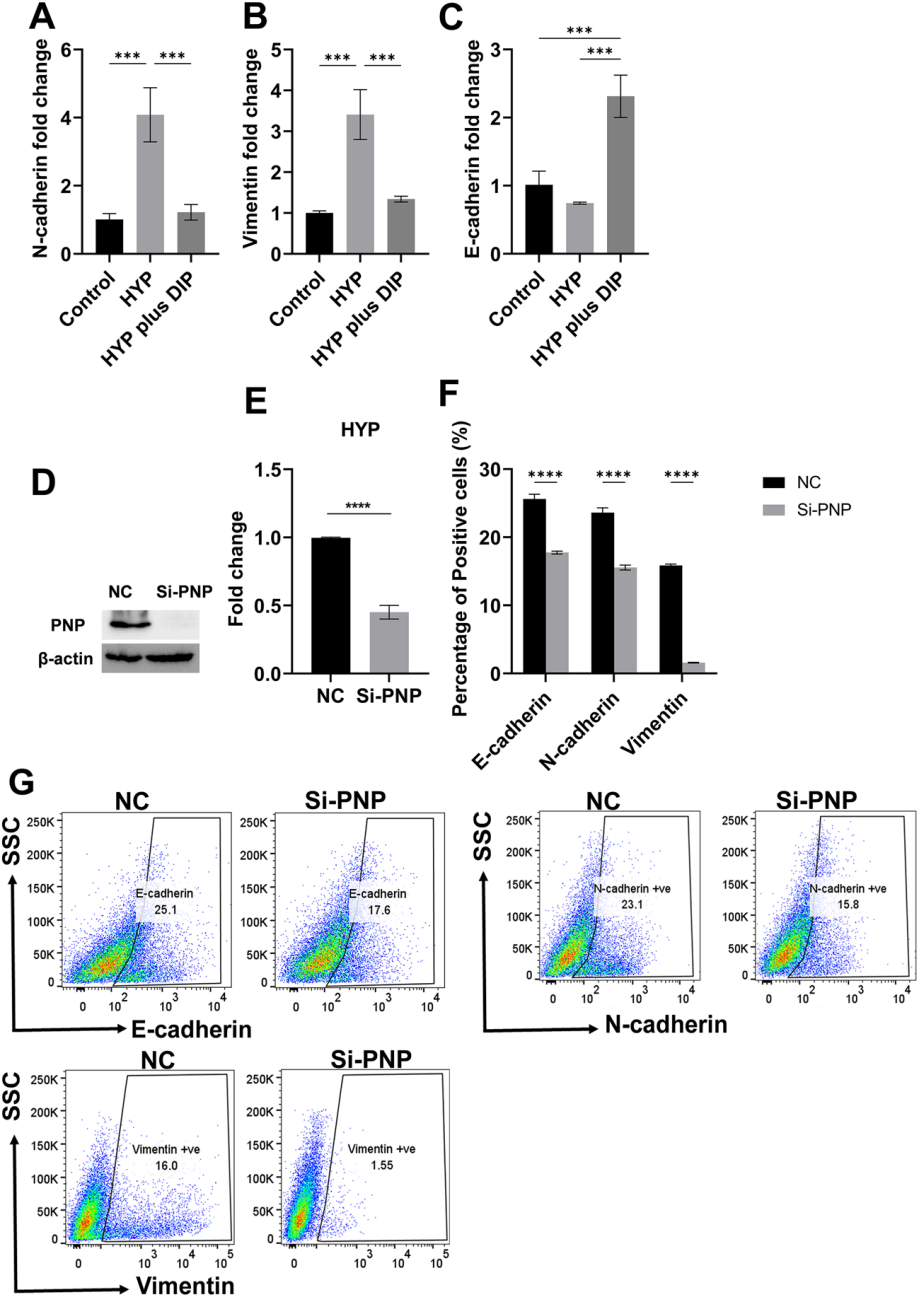
Inhibition of hypoxanthine uptake inhibited hypoxanthine-induced EMT effect. Gene expression analysis of N-cadherin, vimentin and E-cadherin in MCF-7 cells treated with hypoxanthine only (0.1 μM) or hypoxanthine plus dipyridamole (hypoxanthine plus DIP) for testing the expression of (**A**) N-cadherin, (**B**) vimentin and (**C**) E-cadherin. (**D**) PNP protein expression in MDA-MB-231 cells transfected with PNP or negative control (NC) siRNA. (**E**) Hypoxanthine fold change in MDA-MB-231 cells transfected with si-PNP compared to NC. (**F** and **G**) Protein expression of E-cadherin, N-cadherin, and vimentin in MDA-MB-231 cells transfected with PNP siRNA using flow cytometry. Data were presented as mean ± SEM (n = 3). Data were analyzed using one-way ANOVA and Tukey’s multiple comparison test or unpaired t-test. *P* value ≤ 0.05 was considered significant. * Reveals that *P* value < 0.05, ** reveals that *P* value < 0.01, *** reveals that *P* value < 0.001, **** reveals that *P* value < 0.0001. Original blots are presented in Fig. S9.

It has been previously reported that the cellular uptake of hypoxanthine occurs via equilibrative nucleoside transporter (ENT), particularly ENT2^13^. Dipyridamole can inhibit hypoxanthine uptake via the inhibition of ENT2^14,15^. To confirm the hypoxanthine-induced EMT activity, MCF-7 cells were treated for 24 h with either hypoxanthine alone (0.1 µM), or dipyridamole (10 µM) plus hypoxanthine (0.1 µM) (Fig. 6). MCF-7 cells treated with hypoxanthine alone significantly (*P* < 0.001) increased the expression of N-cadherin and vimentin by 4 and 3.4 folds, respectively, while decreasing the mRNA level of E-cadherin by 0.74 folds (Fig. 6A–C). On the other hand, the fold change in the gene expression of N-cadherin and vimentin in MCF-7 cells treated with a combination of dipyridamole and hypoxanthine was 1.22 and 1.3 folds, respectively (Fig. 6A, B). E-cadherin expression was increased when MCF-7 cells were treated with combination of dipyridamole and hypoxanthine by 2.3 folds (Fig. 6C). These results further confirmed the role of hypoxanthine in inducing EMT effect on the less metastatic cells and highlighted the possible modulation of metastatic microenvironment of cancer cells by using hypoxanthine-uptake inhibitors.

### Genetic knockdown of *PNP* significantly reduced hypoxanthine levels in MDA-MB-231 cells but differently affected the expression of EMT markers

To identify the best target that can be used to inhibit hypoxanthine-induced EMT effect, in silico target prediction using ChEMBL database was conducted. The results predicted 5 enzymes and two transporters; out of the 5 enzymes, purine nucleoside phosphorylase (PNP) showed > 50% probability followed by hypoxanthine–guanine phosphoribosyl transferase (HGPRT) (16%), fructose-1,6-bisphosphate aldolase (15%), and aldehyde dehydrogenase 1A1 (13%).

With the highest probability of PNP, its association with metastasis was investigated. MDA-MB-231 cells express a higher level of PNP than MCF-7 cells (Unpublished data). To validate the involvement of PNP in the biosynthesis of hypoxanthine^16^, MDA-MB-231 cells were transfected with siRNA against *PNP*. Transfection with *PNP* siRNA resulted in significant inhibition of PNP protein expression compared to cells transfected with siRNA negative control (Figs. 6D and S9). The level of hypoxanthine was then measured using xanthine/hypoxanthine assay. Interestingly, *PNP* knockdown reduced the level of hypoxanthine by 0.5 folds (Fig. 6E). Notably, Flow cytometry analysis showed significant (*P* < *0.0001*) decrease in the expression of E-cadherin, N-cadherin, and vimentin by 0.6, 0.6, and 0.006 folds, respectively (Fig. 6F, G).

### Hypoxanthine induced the EMT activity by a combination of effectors

#### Hypoxanthine-treated MCF-7 cells displayed a significant EMT effect by increasing the expression of Snail transcription factor

**Figure 7:**
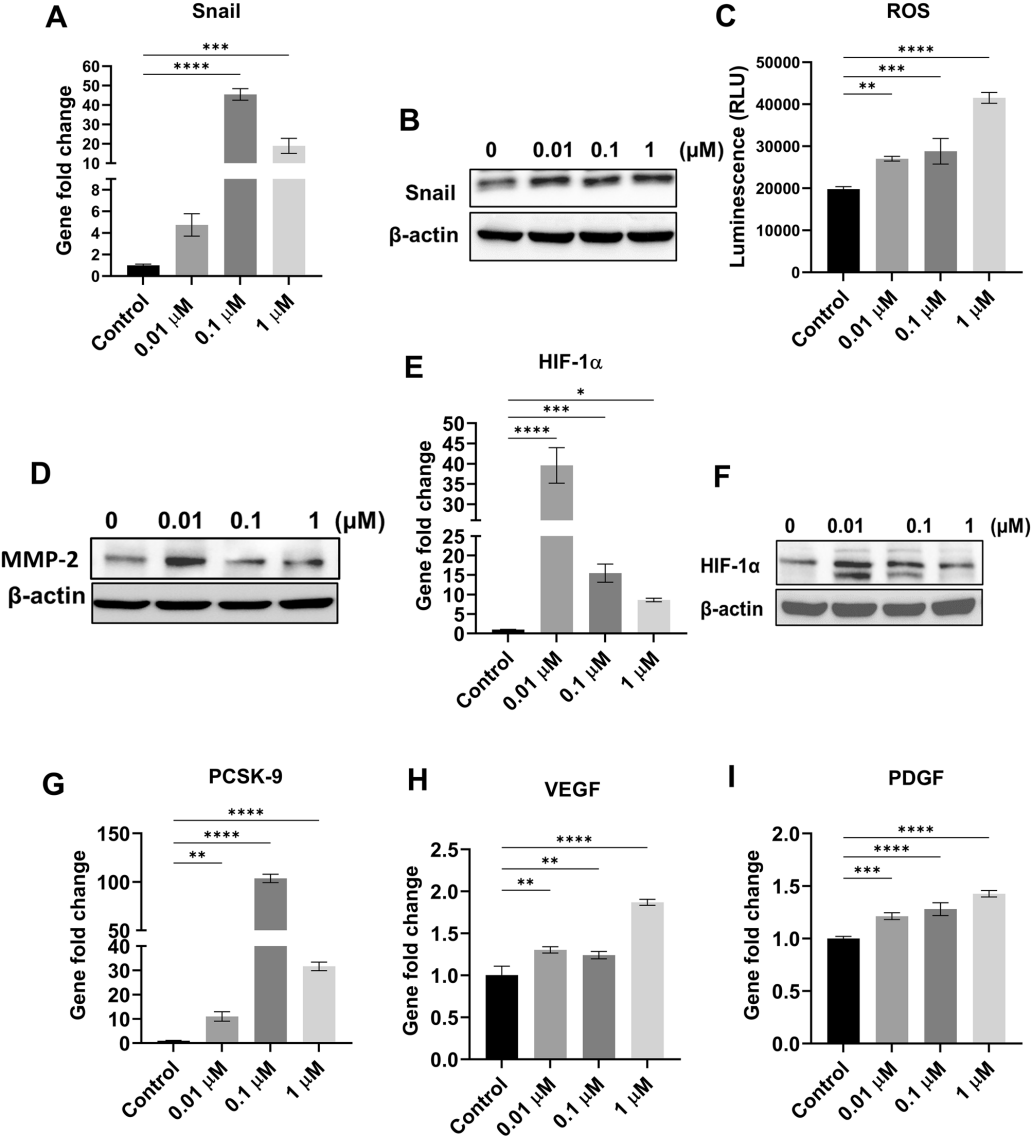
Hypoxanthine-associated mechanisms to induce EMT, migration and invasion in MCF-7 cells. (**A**) Snail gene expression analysis using qRT-PCR. (**B**) Snail protein expression using western blot. (**C**) The luminescence of H_2_O_2_ (ROS) released from MCF-7 cells treated with different concentrations of hypoxanthine. (**D**) MMP-2 protein expression using western blot. (**E**) HIF-1α gene expression analysis using qRT-PCR. (**F**) HIF-1α protein expression using western blot. (**G**) PCSK-9 gene expression. (**H**) VEGF gene expression. (**I**) PDGF gene expression analysis using qRT-PCR. Data were presented as mean ± SEM (n = 3). The data were analyzed using one-way ANOVA) and Tukey’s multiple comparison test. *P* value ≤ 0.05 was considered significant. * Reveals that *P* value < 0.05, ** reveals that *P* value < 0.01, *** reveals that *P* value < 0.001, **** reveals that *P* value < 0.0001. Original blots are presented in Figs. S10 and S11.

Snail *(SNAI1*) is a zinc‐finger transcription factor and a member of large superfamily known as *SNAI* that is essential for cell differentiation and survival^17^. Snail induces EMT by suppressing the transcription of E-cadherin^17^. To identify the mechanism by which E-cadherin gene expression was repressed, the mRNA level of Snail transcription factor was measured. The results indicated that hypoxanthine at 0.1 and 1 µM significantly increased *Snail* gene expression by 45 (*P* < 0.0001) and 18 (*P* < 0.001) folds, respectively (Fig. 7A). To further validate our data, a western blot was conducted. Expectedly, a significant increase in Snail protein abundance in MCF-7 cells by 1.2 folds was observed following treatment with 0.01, 0.1, and 1 µM hypoxanthine (Figs. 7B and S10). Collectively, these findings indicated that hypoxanthine promoted an aggressive phenotype of MCF-7 breast cancer cells and induced EMT potentially by increasing the expression of Snail transcription factor.

#### Hypoxanthine induced the production of reactive oxygen species (ROS) and increased the expression of MMP-2, HIF-1α, and PCSK9

To further investigate the mechanism by which hypoxanthine can induce pro-metastatic effects, the ability of hypoxanthine to induce the production of ROS was investigated; since hypoxanthine is a marker of hypoxia^18^ and ROS is produced in response to hypoxia^19^ and hypoxic cells partially pushed toward EMT^20^. Hypoxanthine-treated MCF-7 cells showed an increase in the production of H_2_O_2_ by 1.36, 1.45, and 2 folds at 0.01, 0.1, and 1 µM, respectively (Fig. 7C).

H_2_O_2_ causes an increase in the expression of matrix metalloproteinases (MMPs), which are responsible for the degradation of extracellular matrix (ECM) and the induction of cancer cell invasion^21^; therefore, the protein level of MMP-2 was measured in hypoxanthine-treated MCF-7 cells. The results showed significant increase in MMP-2 protein abundance by 1.5 and 1.2 folds at 0.01 and 0.1 µM, respectively (Figs. 7D and S11).

Hypoxia inducible factor-1α (HIF-1α) was reported to play an important role in breast cancer metastasis^22^. Treatment of MCF-7 cells with 0.01, 0.1, and 1 µM hypoxanthine significantly (*P* < 0.0001) increased the gene expression of HIF-1α by 39.5, 15.4, and 8.6 folds, respectively (Fig. 7E). To confirm our results, western blot was conducted to measure the protein level of HIF-1α. Hypoxanthine significantly increased the protein levels by 1.5 and 1.3 folds at 0.01 and 0.1 µM respectively (Figs. 7F and S11).

Several studies have suggested a role of proprotein convertase subtilisin/ kexin type 9 (PCSK9) in cancer biology and metastasis^23^. Dramatic and significant (*P* < 0.0001) increase in the mRNA level of PCSK9 was observed in MCF-7 cells treated with 0.1 µM hypoxanthine (103.5 folds) (Fig. 7G). Hypoxanthine at 0.01 and 1µM also caused significant increase in the expression of PCSK9 by 10.9 (*P* < 0.01) and 31.6 (*P* < 0.0001) folds, respectively (Fig. 7G).

#### Hypoxanthine induced the expression of pro-angiogenic factors

Hypoxanthine-treated MCF-7 cells exhibited an increase in the gene expression of proangiogenic factors including VEGF-A and PDGF (Fig. 7H, I). Hypoxanthine at 0.01, 0.1, and 1 µM caused significant increase in the VEGF-A mRNA level by 1.30 (*P* < 0.01), 1.24 (*P* < 0.01), and 1.87 (*P* < 0.0001), respectively (Fig. 7H). Similarly, the PDGF gene expression was increased by 1.2, 1.3, and 1.4 folds at 0.01, 0.1, and 1 µM, respectively (Fig. 7I).

#### In silico analysis of *PNP* gene expression in breast cancer patients validate the significant metastatic activity of hypoxanthine

**Figure 8:**
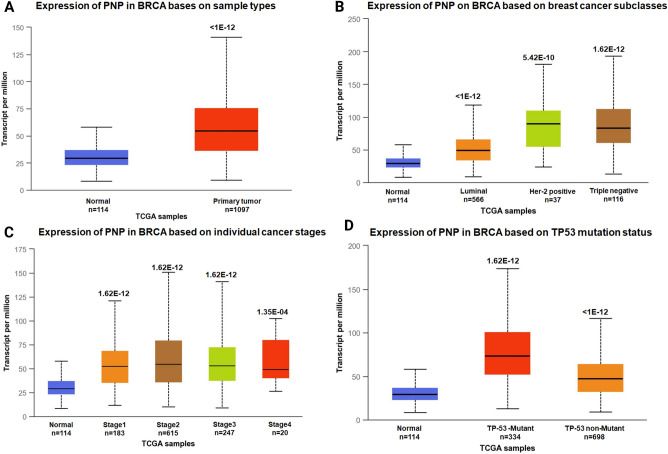
UALCAN analysis for the correlation between *PNP* mRNA expression level and clinicopathological parameters of breast cancer. (**A**) Sample type (normal/primary tumor). (**B**) Breast cancer subclass (luminal, HER2+, and triple negative). (**C**) Cancer stage (stages 1, 2, 3, and 4). (**D**) TP-53 mutation status. BRCA; breast cancer.

To further validate our results, the expression of *PNP* gene was investigated in silico using UALCAN database. *PNP* is significantly high in cancer patients compared to normal individuals (Fig. 8A). Comparison of *PNP* gene expression between major subclasses of cancer showed that *PNP* is significantly higher in Her-2 positive and triple negative subtypes than luminal subtypes, suggesting a role of PNP in cancer aggressiveness (Fig. 8B). Furthermore, *PNP* gene is significantly high in different stages of cancer (Fig. 8C). A significant high expression of *PNP* was observed in patients with TP-53 mutation compared to patients with wild type TP-53, suggesting a positive correlation between PNP and TP-53 mutation (Fig. 8D).

## Discussion

Our results demonstrated that ethyl acetate fraction of MDA-MB-231 conditioned media and its metabolites induced pro-metastatic properties by increasing the gene expression of EMT markers in either a complete or partial EMT fashion.

EMT and its reverse mesenchymal-epithelial transition (MET) are the hallmarks of cancer metastasis^24^. Cells in this phenotype are more aggressive, chemoresistant and able to move collectively in clusters^25^. A significant difference in the metabolomics between the high and less metastatic cells was observed. Most of the secreted metabolites were high in the highly metastatic cells compared to less metastatic cells. In line with our findings, previous report showed that gas chromatography-time of flight-mass spectrometry (GC-TOF–MS) based comparative metabolomic analysis of breast cancer tissues showed a significant difference in the central metabolism between estrogen receptor positive and estrogen receptor negative tissues^26^. Another comparative study between estrogen receptor positive and triple negative breast cancer patients reported differences in 133 metabolites. Majority of them was higher in triple negative breast cancer patients compared to estrogen receptor positive breast cancer patients^27^. Enhanced metabolite excretion can serve as potential biomarkers for identifying highly metastatic cancers^28^.

Hypoxanthine, a naturally occurring purine derivative, is involved in adenosine metabolism and nucleic acids formation by the nucleotide salvage pathway^29^. In the current study, we were able to detect hypoxanthine by ^1^H-NMR in the ethyl acetate fraction of the high-metastatic breast cancer conditioned media but not in the less-metastatic conditioned media. The amount of hypoxanthine inside MDA-MB-231 cells was ~ 3 times higher than in MCF-7 cells. We have found that hypoxanthine is directly responsible for inducing EMT through increasing the expression of Snail, a master regulator of EMT^30^. Consistent with these data, high levels of hypoxanthine were recently identified as biomarker in human melanoma metastasis^31^, lung squamous cell carcinoma metastasis^32^ and prostate cancer aggressiveness^33^. Similarly, the highest level of hypoxanthine was reported in stage IV compared to stage II and III, indicating its role in colorectal cancer progression^34^. These reports may further support the role of hypoxanthine in cancer metastasis.

PNP (EC 2.4.2.1) is responsible for the reversible conversion of inosine to hypoxanthine and guanosine to guanine^35^. As a result, purines and their deoxyribonucleosides are converted to mononucleotides during purine salvage pathway^35^. In silico analysis showed that breast cancer patients exhibited higher expression levels of *PNP*. Similarly, elevated levels of PNP were observed in triple negative breast cancer subtypes compared to luminal subtypes, suggesting its association with breast cancer aggressiveness. PNP was found to be associated with colorectal cancer aggressiveness^36^. In our study, genetic knockdown of *PNP* showed significant decrease in the protein expression of mesenchymal markers, in addition to epithelial marker. The decrease in these proteins may be due to decrease in hypoxanthine level in MDA-MB-231 cells and/ or to the effect of other metabolites. Accumulation of inosine due to PNP inhibition in T-cells was previously reported^37^. Further, inhibition of PNP increased the plasma concentrations of 2′-deoxyguanosine, which in turn leads to accumulation of deoxyguanosine triphosphate (dGTP) within the cells, resulting in cell death^38^. These effects may explain the decrease in E-cadherin in knockdown cells.

During the purine catabolism pathway, hypoxanthine is converted to xanthine, which is then converted to uric acid by xanthine oxidase (XOR). This is associated with the production of ROS as by-product^34^. It has been demonstrated that the loss of XOR expression is associated with aggressiveness in breast cancer^39^. Similarly, XOR was found to be differentially expressed in breast cancer tissues and its loss is associated with breast cancer aggressiveness and poor prognosis^40^. Hypoxanthine has long been recognized as a hypoxia biomarker^18^. Breast cancer cells that are distant from functioning blood vessels have significantly reduced oxygen levels, resulting in a hypoxic microenvironment^41^. Breast cancer cells respond to hypoxia by increasing the levels of hypoxia-inducible factors (HIFs), which are associated with EMT, angiogenesis, glucose utilization, oxidative stress resistance, proliferation, resistance to apoptosis, invasion, and metastasis. In our study, treatment of MCF-7 cells with hypoxanthine increased the release of H_2_O_2_. It was demonstrated that H_2_O_2_ causes an increase in MMPs expression, which can degrade the extracellular matrix (ECM) and hence can enhance the process of cancer cells invasion and migration^21^. We have observed that hypoxanthine increased the protein expression of MMP-2 in MCF-7 cells, confirming the role of hypoxanthine in invasion and metastasis. HIFs play a key role in the cellular response to oxidative stress^19^. Since the molecular oxygen is required for the formation of ROS, it has been assumed that ROS may be involved in the response to hypoxia^19^. It has been reported that hypoxic cells are partially pushed towards EMT by inducing Snail expression^20^. We demonstrated that hypoxanthine-treated MCF-7 cells exhibited an induced gene expression of HIF-1α.

Passive transport of hypoxanthine occurs via ENT1 and ENT2 that allow its diffusion across concentration gradients^13^. ENT is inhibited by dipyridamole in a competitive manner^15^. In the current study, treatment of MCF-7 cells with dipyridamole plus hypoxanthine significantly inhibit hypoxanthine-induced EMT effect, indicating significant decrease in hypoxanthine uptake. Angiogenesis is a process of development of new blood vessels, which is an important step for breast cancer dissemination and metastasis^42^. The angiogenesis process is stimulated by a variety of physiological and pathological factors, with hypoxia being the primary stimulus. The most important angiogenic factor is VEGF-A which is primarily regulated by HIF-1 transcriptional factor^43^. Hypoxanthine was found to significantly increase the expression of VEGF-A and PDGF-B, indicating its role in angiogenesis.

**Figure 9:**
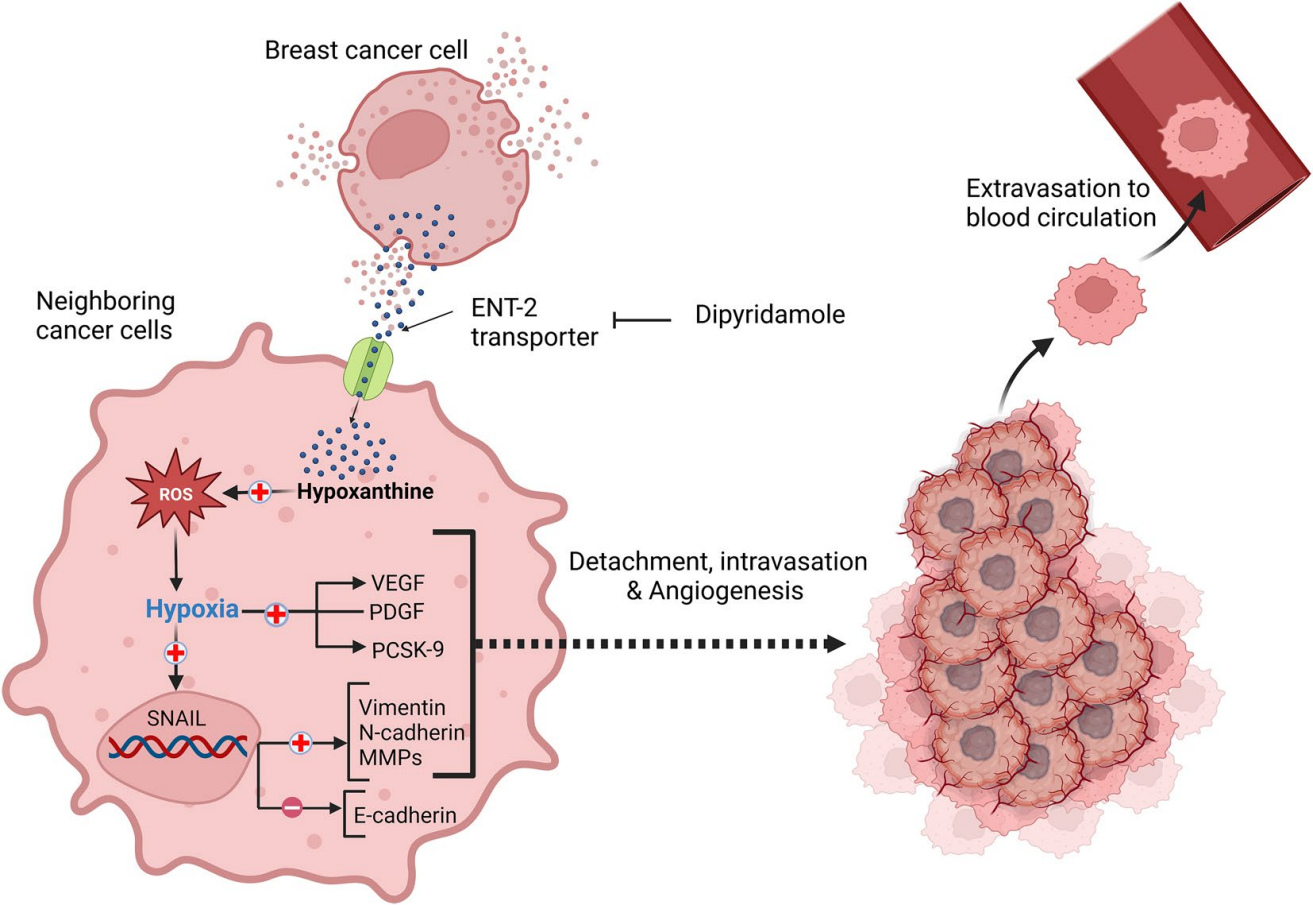
Hypothetical model for the mechanistic effect of hypoxanthine on invasion and metastasis of MCF-7 cells. Hypoxanthine is uptake through equilibrative nucleoside transporter (ENT2). Hypoxanthine induces hypoxia and the release of reactive oxygen species (ROS). Hypoxia induces the expression of HIF-1α, which activates Snail transcription factor. Snail in turn, represses the expression of E-cadherin. Hypoxanthine also increases the expression of N-cadherin, vimentin, MMP-2, PCSK-9, and VEGF, and PDGF, resulting in EMT, pro-angiogenesis and invasion.

PCSK9 belongs to the proprotein convertase family of secretory serine proteases. A key function of PCSK9 is the control of the protein levels of the low-density lipoprotein receptor (LDLR) as it enhances its internalization and degradation in endosomal/lysosomal compartments, thereby, regulating cholesterol homeostasis^44^. It has been found that breast cancer cells overexpress PCSK9 and have a high cholesterol content^44^. Studies have shown that hypercholesterolemia accelerates the progression of estrogen-positive breast cancer and increases resistance to hormonal therapy^45^. Further, cholesterol was found to be associated with breast cancer proliferation and aggressive potential^46^. Increased levels of PCSK9 in gastric cancer tissue were correlated with gastric cancer progression and poor prognosis^47^. HIF-1α and ROS stimulators were reported to increase the expression of PCSK9 in cardiomyocytes, while ROS inhibitors suppress PCSK9 expression suggesting a cross talk between ROS and PCSK9^48^. In our study, hypoxanthine was found to substantially increase the levels of PCSK9 suggesting another mechanism by which hypoxanthine can induce metastasis. Collectively, these findings suggest a mechanism by which hypoxanthine can induce pro-metastatic effect represented in our Model (Fig. 9).

The results of this study provide further evidence that altered metabolism is not only a consequence of tumor growth, but metabolites play an essential role in driving cancer pathogenesis and progression.

## Materials and methods

### Materials

Dulbecco’s modified Eagle’s medium (DMEM) (# 6429), Fetal bovine serum (FBS) (# F9665), Dulbecco’s phosphate buffered saline (PBS) (# 8537), penicillin/streptomycin (# P4458), hexane, ethyl acetate, dichloromethane, methanol, xanthurenate (# D120804), sodium pyruvate (# P2256), inosine (# I4125), uridine (# U3750), betaine (# B2629), β-alanine (# A 9920), xanthine (# X7375), hypoxanthine (# H9636), dipyridamole (# D9766), 3-(trimethylsilyl)-1- propane sulfonic acid-d6 sodium salt (# 178837) and methanol-d4 (# 151947) were purchased from Sigma-Aldrich Co. (St. Louis, MI, USA). Recombinant APC anti-N cadherin antibody (# ab275670), HIF-1α rabbit mAb (# ab179483) and xanthine/hypoxanthine assay kit (# ab155900) were purchased from Abcam (Cambridge, UK). ROS-Glo™ H_2_O_2_ Assay kit (# G8820) was purchased from Promega (Madison, USA). Pierce BCA protein assay kit (# 23227), Snail polyclonal antibody (# PA5-119607), lipofectamine RNAiMAX reagent (# 13778150), silencer™ select SiRNA against *PNP* (# 4390824) (s9656), silencer™ Select negative control siRNA (# 4390844) and RNA extraction kit (# 12183020) were purchased from Thermo Fisher Scientific (Waltham, Massachusetts, USA). SensiFAST™ SYBR Hi-ROX Kit (# BIO-92005) and SensiFAST™ cDNA synthesis kit (# BIO-65053) were purchased from Bioline (Germany). MMP-2 rabbit mAb (# 13132), β-actin rabbit mAb (# 4970), anti-rabbit IgG, HRP-linked antibody (# 7074S), anti-mouse IgG, HRP-linked Antibody (# 7076), E-cadherin rabbit mAb (Alexa Fluor® 488 Conjugate) (# 3199) and vimentin rabbit mAb (Alexa Fluor® 488 Conjugate) (#9854) were purchased from cell signaling technology (Massachusetts, USA). Clarity western ECL substrate detection system (# 170–5060) was purchased from Biorad (California, USA). PNP antibody mouse mAB (# MAB6486) was purchased from R&D systems (Minneapolis, Minnestoa, USA**)**.

### Cell lines and culture conditions

MCF-7 breast cancer cell line was obtained from cell lines service (CLS; Germany). MDA-MB-231 breast cancer cell line was obtained from European Collection of Authenticated Cell Cultures (ECACC; Salisbury, UK). The cells were authenticated and tested negative for mycoplasma. These cells were cultured in Dulbecco’s Modified Eagle Medium (DMEM) growth media supplemented with 10% fetal bovine serum (FBS) and 1% penicillin/ streptomycin at 37 °C in a humidified atmosphere of 5% CO_2_. Three biological replicates from each cell line were prepared.

### Metabolite extraction

5 × 10^5^ Cells from MCF-7 or MDA-MB-231 (passage # 15) were seeded in a 75 cm^2^ tissue culture flask (10 flasks) and incubated for 48 h. At the time of extraction, culture media of a total number of cells 20 × 10^6^, were collected and subjected to solvent fractionation using hexane, dichloromethane (DCM) and ethyl acetate. Media without cells were fractionated similarly and employed as negative control. To measure the level of a metabolite inside the cells, the cell pellets were extracted using methanol. The extraction procedure was performed in triplicates. All organic solvents were completely evaporated, and the residues were dissolved in 0.001% dimethyl sulphoxide (DMSO), followed by dilution in culture media prior to investigation.

### Cell culture treatment

To test the effect of extracted extracellular metabolites, MCF-7 or MDA-MB-231 cells were seeded and treated for 48 h with either 100 µg/ml hexane, DCM or ethyl acetate fractions extracted from MCF-7 or MDA-MB-231 conditioned media. To evaluate the effect of the highly expressed metabolites in the ethyl acetate fraction of MDA-MB-231 cells, MCF-7 cells were plated and treated daily for 48 h with either 1, 10, or 100 µM xanthurenate, pyruvate, inosine, uridine, betaine, β-alanine, xanthine, or hypoxanthine.

To study the effect of dipyridamole, MCF-7 cells were seeded in 6-well-plate and treated with either hypoxanthine alone (0.1 µM), or dipyridamole (10 µM) plus hypoxanthine (0.1 µM) for 24 h.

### Quantitative real-time polymerase chain reaction (qRT-PCR)

Invitrogen™ Pure link™RNA Mini Kit was used for RNA extraction following the manufacturer’s protocol. The resulting RNA was used for cDNA synthesis according to SensiFAST™ cDNA Synthesis Kit. Primers were purchased from Microgen (Korea) and were described in Table S1. Real-time PCR was conducted on Quant Studio 3 (ThermoScinetific) using SensiFAST™ SYBR Hi-ROX kit. All samples were amplified in triplicates. The average threshold cycle (Ct) values for the genes were obtained from each reaction and the expression was quantified using the 2^(−ΔΔC(T))^ relative method.

### Western blot analysis

RIPA buffer (25mM Tris/HCl pH 7.6, 150mM NaCl, 1% NP-40, 1% sodium deoxycholate, 0.1% SDS) was used to lyse the mammalian cells. The protein content of each sample was quantified using Pierce BCA protein assay kit following the manufacturer’s instructions. A volume equivalent to 30 µg of total protein was separated on 10% sodium dodecyl sulphate-poly acrylamide gel electrophoresis (SDS-PAGE). The separated proteins were electrophoretically transferred to nitrocellulose membrane, which was then blocked in 5% skimmed milk. The membrane was probed with primary antibodies, listed as ‘target protein’ HIF-1α (1:500), Snail (1:500), MMP-2 (1:500), PNP (1:1000) and β-actin (1:2000) overnight at 4 °C. When the membrane probed with more than one antibody, mild stripping buffer was used before the addition of second antibody. Membranes were then incubated with anti-rabbit or anti-mouse IgG horseradish peroxidase-conjugated (HRP) linked (1:1000) for 1h at room temperature. The signals were developed using Clarity western ECL substrate detection system.

### Flow cytometry analysis

The expression of cell surface markers, E-cadherin, N-cadherin, and vimentin of the metabolite-treated MCF-7 cells was evaluated by flow cytometry. Briefly, cells were harvested and washed with cold fluorescence activated cell sorting (FACS) buffer (PBS, 2% FBS, 0.1% (10 mM) sodium azide) followed by incubation with E-cadherin rabbit monoaclonal antibody (Alexa Fluor® 488 Conjugate), N-cadherin rabbit monoaclonal antibody or vimentin rabbit monoaclonal antibody (Alexa Fluor® 647 Conjugate). Unstained cells were employed as control. Flow cytometry analysis was adapted from our previously described method^49^. Cells were incubated for 45 min at 4 °C, washed with FACS buffer and the secondary antibody (Alexa Fluor® 488 conjugate) was added to the samples and incubated with the N-cadherin rabbit mAB antibody. Afterwards, the samples were washed twice with ice cold PBS and subsequently analyzed by flow cytometry using BD FACSAria III (BD Biosciences, San Jose, CA, USA). The viable cells were selected, and the gating was adjusted according to unstained cells. Data were acquired by BD FACS Diva software (BD Biosciences, San Jose, CA, USA) using standard fluidics, optical and electronic configuration and then analyzed using FlowJo software.

### *PNP* gene silencing

MDA-MB-231 cells were seeded in a 6-well plate at a density of 2 × 10^5^ cells/well in antibiotic-free medium. In the next day, cells were transfected with siRNA using lipofectamine RNAiMAX reagent (# 13778150, ThermoScientific) following manufacturer’s recommendations. The cells were transfected with 50 nM of Silencer™ select siRNA against *PNP* (# 4390824 (s9656), ThermoScientific). Silencer™ Select negative control siRNA (# 4390844, ThermoScientific) was employed as negative control. The cells were harvested after 72 h of transfection for further analysis.

### Measurement of xanthine/hypoxanthine concentration

Xanthine/ hypoxanthine levels were quantified using xanthine/hypoxanthine assay kit. The assay was conducted according to manufacturer’s instructions. For metabolites extraction, 3 × 10^5^ MDA-MB-231 cells were homogenized in 100 μl ice-cold assay buffer and the resulting homogenates were deproteinized using perchloric acid/KOH deproteinization protocol. Xanthine/hypoxanthine was specifically oxidized by a xanthine enzyme mix provided in the kit to form an intermediate, which then reacted with a developer and a probe to form a product. The absorbance was measured at 570nm using Multiskan go microplate spectrophotometer (Thermo Fisher Scientific, Waltham, MA, USA).

#### Detection of reactive oxygen species (ROS)

The level of hydrogen peroxide (H_2_O_2_) was measured using ROS-GIO H_2_O_2_ assay kit (# G8820, Promega) following the manufacturer’s instructions and the luminescence was detected using Multiskan go microplate spectrophotometer (ThermoScientific).

#### NMR spectroscopy

Equal weights of fraction, extracellular or intracellular metabolite extracts were mixed with 3-(trimethylsilyl)-1- propane sulfonic acid-d6 sodium salt (DSS-d6, dissolved in methanol-d4, 10 mM) as an internal reference to final concentration of 1 mM. Three biological replicates from each sample were prepared and 700 μL of each sample was transferred to 5 mm NMR tubes prior to ^1^H-NMR spectroscopy analysis according to an adapted method previously published^50^. Briefly, ^1^H-NMR were performed using Bruker Avance III HD NMR spectroscopy (Bruker Biospin GmbH, Karlsruhe, Germany) operating at 500 MHz and 298 K (25 °C) temperature using a standard 90° pulse sequence (zg) with a 4 min and 44s acquisition time, 1s relaxation delay, 64 scans, 64 k data points, and 10,000 Hz spectral width (20 ppm). Each free-induction decay was zero-filled to 64 k points and multiplied by a 0.3 Hz exponential function prior to Fourier transformation. The raw data set can be accessed at https://www.ebi.ac.uk/metabolights/MTBLS7764. Username: U19106034@sharjah.ac.ae and password: Hala1542020.

Metabolite annotation was achieved via fitting with its reference spectrum from the library of Chenomx NMR suite (v. 8.6, Edmonton, AB, Canada) after phase and baseline correction. Clusters that appear green in the Cluster Navigator in Chenomx database were considered 'matched'. Human Metabolome Database (HMDB) and previously published data were used to further verify the annotation. Quantification of metabolites was conducted using ChenomX NMR Suite 8.6. after matching the shape and position of a compound signature in the software to the shape of our spectrum (peak-based fit style). Compound concentrations were measured by determining the height of each compound signature that best matches the peak heights in our spectrum. When the height of a compound is adjusted, the heights of all peaks and clusters corresponding to the compound were scaled proportionately. The internal standard DSS (1 mM) was used as an internal reference to calibrate the concentration and chemical shifts of the analytes. The calculated metabolite concentration is the concentration released from 20 million cells. The fold change was calculated by dividing the concentration of metabolite in MCF-7 by the concentration of metabolite in MDA-MB-231 cells. The metabolites with corresponding concentrations were subjected to multivariate analysis.

The metabolomic pattern between the different samples was interpreted using metabolite set enrichment analysis (MSEA). MSEA tests the enrichment of metabolites in a metabolic pathway in comparison to the total annotated metabolites in the same pathway. MSEA was performed using free platform MetaboAnalyst 5.0^51^.

#### Transwell migration and invasion assay

For migration assay, 1 × 10^4^ MCF-7 cells were suspended in serum-free medium and plated in 24-well cell culture inserts with transparent PET membrane (8 μm pore size) (Corning) without Matrigel. For cell invasion assay, 1 × 10^4^ cells were resuspended in serum-free medium and seeded in the upper chamber coated with Matrigel (# 354480, Corning). Medium containing 10% FBS (650 mL) was added to the lower chamber. After incubation for 20 h, the cells in the lower chamber were fixed with methanol and stained with 0.2% crystal violet. Olympus DP-74 camera (Tokyo, Japan) was used to capture crystal violet-stained cells and ImageJ (version 1.53e) was used for quantification.

#### In silico prediction of hypoxanthine biosynthesis target using ChEMBL bioactivity database

In silico target prediction for the hypoxanthine biosynthesis was developed using bioactivity data extracted from the ChEMBL database^52^.

#### Analysis of *PNP* gene in breast cancer based on UALCAN database

Using UALCAN (http://ualcan.path.uab.edu), transcriptional level of PNP is compared between normal and tumor samples as well as between different types and stages of breast cancer. In addition, the relationship between transcriptional level and clinical parameters was also investigated. The screening conditions set in this study are: “Gene: PNP”; “Cancer Type: Breast invasive carcinoma”; “Data Type: TCGA dataset”.

#### LC–MS/MS analysis

To measure the level of HYP in MCF-7 and MDA-MB-231 cells and their conditioned media, HYP was extracted from the cell pellet using methanol and from the conditioned media using ethyl acetate. Dried samples were dissolved in 0.1% formic acid. 50 µl of each sample was spiked with HYP at 2 µM. Unknown concentrations of HYP were calculated using an established calibration curve (1, 1.25, 2.5, 5 µM). The separation and mass spectrometry analysis of HYP was conducted using a Waters® Acquity H-Class UPLC®-tandem triple-quadrupole mass spectrometry (TQD) (Milford, MA, USA). The H-Class UPLC® system includes Acquity sample manager and Acquity quaternary solvent manager. TQD was equipped with electrospray ionization (ESI) probe. The chromatographic separation was achieved using 0.1% formic acid in acetonitrile (A) and 0.1% formic acid in water (B) at a flow rate of 0.2 ml/min in an isocratic elution of 90% A. HYP was eluted on ZORBAX RRHD Eclipse plus C18 (50 × 2.1 mm, 1.8 µm) column (Agilent, California, USA) maintained at 25 °C and 6000 psi. Total sample run time was 1.5 min. The injection volume was 10 µl and the temperature of autosampler was kept at 20 ± 3 °C. Mass spectrometry parameters were maintained as follows: cone gas flow; 6 L/h, nitrogen gas flow; 600 L/h, capillary voltage; 2.3 kV, the ion source temperature; 150 °C, and the desolvation temperature was set at 300 °C. The cone voltage and collision energy of HYP were set at 38 and 22 V, respectively. Quantification was performed using multiple reaction monitoring (MRM) mode by monitoring the transition ions of 136.92 m/z as parent to 109.96 m/z as fragment with the cone voltage of 38 and collision energy of 22.

#### Statistical analysis

Tables composed of metabolite names, sample names and concentrations from all replicates were imported into MetaboAnalyst 5.0 platform for multivariate statistical analysis including hierarchical cluster analyses (HCA), principal component analysis (PCA), partial least squares-discriminate analysis (PLS-DA), and variable importance in projection scores (VIP)^51^. VIP values higher than 1.00 were considered significant. Venn diagrams were generated using R programming language (version 3.6.2).

For univariate analysis, GraphPad Prism was used. Fold changes in concentration were calculated as a ratio of average metabolite concentrations between MDA-MB-231 and MCF-7 cells. For *in-vitro* experiments, the means ± SEM of three independent experiments and the unpaired student t-test or one-way analysis of variance (ANOVA) were used. Tukey’s multiple comparison tests were employed to compare between the expression of different genes.

### Supplementary Information

Supplementary Information.

## Data Availability

All data generated or analyzed during this study are included in this published article and its supplementary information files.

## References

[1] WHO. Estimated number of deaths in 2020, world, Females, all ages. https://gco.iarc.fr/today/online-analysis-pie?v=2020&mode=cancer&mode_population=continents&population=900&populations=900&key=total&sex=2&cancer=39&type=1&statistic=5&prevalence=0&population_group=0&ages_group%5B%5D=0&ages_group%5B%5D=17&nb_items=7&group_cancer=1&include_nmsc=1&include_nmsc_other=1&half_pie=0&donut=0 (2023).

[2] Lambert AW, Pattabiraman DR, Weinberg RA (2017). Emerging biological principles of metastasis. Cell.

[3] Giancotti FG (2013). Mechanisms governing metastatic dormancy and reactivation. Cell.

[4] Thiery JP, Sleeman JP (2006). Complex networks orchestrate epithelial–mesenchymal transitions. Nat. Rev. Mol. Cell Biol..

[5] Hanahan D, Weinberg RA (2011). Hallmarks of cancer: The next generation. Cell.

[6] Wei Q, Qian Y, Yu J, Wong CC (2020). Metabolic rewiring in the promotion of cancer metastasis: Mechanisms and therapeutic implications. Oncogene.

[7] Colvin H (2016). Oncometabolite D-2-hydroxyglurate directly induces epithelial-mesenchymal transition and is associated with distant metastasis in colorectal cancer. Sci. Rep..

[8] Sciacovelli M (2016). Fumarate is an epigenetic modifier that elicits epithelial-to-mesenchymal transition. Nature.

[9] Wang H, Chen Y, Wu G (2016). SDHB deficiency promotes TGFβ-mediated invasion and metastasis of colorectal cancer through transcriptional repression complex SNAIL1-SMAD3/4. Transl. Oncol..

[10] Gomes AP (2020). Age-induced accumulation of methylmalonic acid promotes tumour progression. Nature.

[11] Soliman SS (2020). Effective targeting of breast cancer cells (MCF7) via novel biogenic synthesis of gold nanoparticles using cancer-derived metabolites. PLoS One.

[12] Teoh ST, Lunt SY (2018). Metabolism in cancer metastasis: Bioenergetics, biosynthesis, and beyond. Wiley Interdiscip. Rev. Syst. Biol. Med..

[13] Senyavina N, Tonevitskaya S (2015). Effect of hypoxanthine on functional activity of nucleoside transporters ENT1 and ENT2 in caco-2 polar epithelial intestinal cells. Bull. Exp. Biol. Med..

[14] Marshman E, Taylor GA, Thomas HD, Newell DR, Curtin NJ (2001). Hypoxanthine transport in human tumour cell lines. Relationship to the inhibition of hypoxanthine rescue by dipyridamole. Biochem. Pharmacol..

[15] Schaper W (2005). Dipyridamole, an underestimated vascular protective drug. Cardiovasc. Drugs Ther..

[16] Xia Y (2021). GABA transporter sustains IL-1β production in macrophages. Sci. Adv..

[17] Nieto MA (2002). The snail superfamily of zinc-finger transcription factors. Nat. Rev. Mol. Cell Biol..

[18] Saugstad OD (1975). Hypoxanthine as a measurement of hypoxia. Pediatr. Res..

[19] Kietzmann, T. & Görlach, A. *Seminars in Cell & Developmental Biology* 474–486 (Elsevier). 10.1016/j.semcdb.2005.03.010 15905109

[20] Lundgren K, Nordenskjöld B, Landberg G (2009). Hypoxia, Snail and incomplete epithelial–mesenchymal transition in breast cancer. Br. J. Cancer Res..

[21] Belkhiri A, Richards C, Whaley M, McQueen SA, Orr FW (1997). Increased expression of activated matrix metalloproteinase-2 by human endothelial cells after sublethal H_2_O_2_ exposure. Lab. Investig..

[22] Liu Z-J, Semenza GL, Zhang H-F (2015). Hypoxia-inducible factor 1 and breast cancer metastasis. J. Zhejiang Univ. Sci. B.

[23] Sun X (2012). Proprotein convertase subtilisin/kexin type 9 deficiency reduces melanoma metastasis in liver. Neoplasia.

[24] Jolly MK (2015). Implications of the hybrid epithelial/mesenchymal phenotype in metastasis. Front. Oncol..

[25] Lüönd F (2021). Distinct contributions of partial and full EMT to breast cancer malignancy. Dev. Cell.

[26] Budczies J (2013). Comparative metabolomics of estrogen receptor positive and estrogen receptor negative breast cancer: Alterations in glutamine and beta-alanine metabolism. J. Proteomics.

[27] Kanaan YM (2014). Metabolic profile of triple-negative breast cancer in African-American women reveals potential biomarkers of aggressive disease. Cancer Genomics Proteomics.

[28] Melo LMN, Lesner NP, Sabatier M, Ubellacker JM, Tasdogan A (2022). Emerging metabolomic tools to study cancer metastasis. Trends Cancer.

[29] Wishart DS (2022). HMDB 5.0: The human metabolome database for 2022. Nucleic Acids Res..

[30] De Herreros AG, Peiró S, Nassour M, Savagner P (2010). Snail family regulation and epithelial mesenchymal transitions in breast cancer progression. J. Mammary Gland Biol. Neoplasia.

[31] Kosmopoulou M (2020). Human melanoma-cell metabolic profiling: Identification of novel biomarkers indicating metastasis. Int. J. Mol. Sci..

[32] Lee H (2021). Integrative metabolomic and lipidomic profiling of lung squamous cell carcinoma for characterization of metabolites and intact lipid species related to the metastatic potential. Cancers.

[33] Dudka I (2020). Comprehensive metabolomics analysis of prostate cancer tissue in relation to tumor aggressiveness and TMPRSS2-ERG fusion status. BMC Cancer.

[34] Naes SM, Ab-Rahim S, Mazlan M, Amir Hashim NA, Abdul Rahman A (2023). Increased ENT2 expression and its association with altered purine metabolism in cell lines derived from different stages of colorectal cancer. Exp. Ther. Med..

[35] Bzowska A, Kulikowska E, Shugar D (2000). Purine nucleoside phosphorylases: properties, functions, and clinical aspects. Pharmacol. Ther..

[36] Sanfilippo O (1994). Relationship between the levels of purine salvage pathway enzymes and clinical/biological aggressiveness of human colon carcinoma. Cancer Biochem. Biophys..

[37] Wang T (2020). Inosine is an alternative carbon source for CD8^+^-T-cell function under glucose restriction. Nat. Metab..

[38] Alonso R (2007). The purine nucleoside phosphorylase inhibitor forodesine (BCX-1777) is a potent cytotoxic agent and has synergistic activity with bendamustine in chronic lymphocytic leukemia (CLL) irrespective of ZAP-70 levels and p53 status. Blood.

[39] Fini MA (2008). Migratory activity of human breast cancer cells is modulated by differential expression of xanthine oxidoreductase. J. Cell Biochem..

[40] Linder N (2005). Down-regulated xanthine oxidoreductase is a feature of aggressive breast cancer. Clin. Cancer Res..

[41] Gilkes DM, Semenza GL (2013). Role of hypoxia-inducible factors in breast cancer metastasis. Future Oncol..

[42] Longatto Filho A, Lopes JM, Schmitt FC (2010). Angiogenesis and breast cancer. J. Oncol..

[43] Ferrara N, Gerber H-P, LeCouter J (2003). The biology of VEGF and its receptors. Nat. Med..

[44] Maxwell KN, Fisher EA, Breslow JL (2005). Overexpression of PCSK9 accelerates the degradation of the LDLR in a post-endoplasmic reticulum compartment. Proc. Natl. Acad. Sci..

[45] Kuzu OF, Noory MA, Robertson GP (2016). The role of cholesterol in cancer. Cancer Res..

[46] de Gonzalo-Calvo D (2015). Intratumor cholesteryl ester accumulation is associated with human breast cancer proliferation and aggressive potential: A molecular and clinicopathological study. BMC Cancer.

[47] Xu B (2021). Proprotein convertase subtilisin/kexin type 9 promotes gastric cancer metastasis and suppresses apoptosis by facilitating MAPK signaling pathway through HSP70 up-regulation. Frront. Oncol..

[48] Ding Z (2018). PCSK9 expression in the ischaemic heart and its relationship to infarct size, cardiac function, and development of autophagy. Cardiovasc. Res..

[49] Soliman SS (2022). Novel secreted peptides from *Rhizopus arrhizus* var. *delemar* with immunomodulatory effects that enhance fungal pathogenesis. Front. Microbiol..

[50] Chen L (2020). Metabolic characterisation of eight *Escherichia coli* strains including “Big Six” and acidic responses of selected strains revealed by NMR spectroscopy. Food Microbiol..

[51] Xia J, Wishart DS (2011). Metabolomic data processing, analysis, and interpretation using MetaboAnalyst. Curr. Protoc. Bioinform..

[52] Mayr A (2018). Large-scale comparison of machine learning methods for drug target prediction on ChEMBL. Chem. Sci..

