# Supplementary material for: 1H-NMR metabolomics analysis identifies hypoxanthine as a novel metastasis-associated metabolite in breast cancer
Source: Sci Rep. 2024 Jan 2;14:253. doi: 10.1038/s41598-023-50866-y (PMC10762038; doi:10.1038/s41598-023-50866-y)
Supplement: Supplementary file 1 — Supplementary Information. [file 41598_2023_50866_MOESM1_ESM.docx]

**Supplementary Information**

**^1^H-NMR metabolomics analysis identifies hypoxanthine as a novel metastasis-associated metabolite in breast cancer**

**Sarra B. Shakartalla^1,2,3^, Naglaa S. Ashmawy^1,4,5^, Mohammad H. Semreen^1,10^, Bahgat Fayed^1,6^, Zainab M. Al Shareef^2^, Manju N. Jayakumar^1^, Saleh Ibrahim^1,7^,** **Mohamed Rahmani^1,7,8^, Rania Hamdy^1,9^, and Sameh S.M. Soliman^1,10*^**

^1^Research Institute for Medical and Health sciences, University of Sharjah, P.O. Box 27272, Sharjah, United Arab Emirates
^2^College of Medicine, University of Sharjah, P.O. Box 27272, Sharjah, United Arab Emirates
^3^Faculty of Pharmacy, University of Gezira, P.O.Box. 21111, Wadmedani, Sudan
^4^College of Pharmacy, Gulf Medical University, Ajman P.O. Box 4184, United Arab Emirates

^5^Faculty of Pharmacy, Department of Pharmacognosy, Ain Shams University, P.O. Box 11566-Abbassia, Cairo, Egypt

^6^Chemistry of Natural and Microbial Product Department, National Research Centre, Cairo P.O. Box 12622, Egypt

^7^Center for Biotechnology, Khalifa University, Abu Dhabi^8^College of Medicine and Health Sciences, Khalifa University, Abu Dhabi P.O. Box 127788, United Arab Emirates

^9^Faculty of Pharmacy, Zagazig University, Zagazig P.O. Box 44519, Egypt

^10^College of Pharmacy, University of Sharjah P.O. Box 27272, Sharjah, United Arab Emirates

***Correspondence:**

**Sameh Soliman**

Department of Medicinal Chemistry, College of Pharmacy, University of Sharjah, Sharjah, UAE, Tel: +97165057472, Email: [ssoliman@sharjah.ac.ae](mailto:ssoliman@sharjah.ac.ae)

**Supplementary Figures**


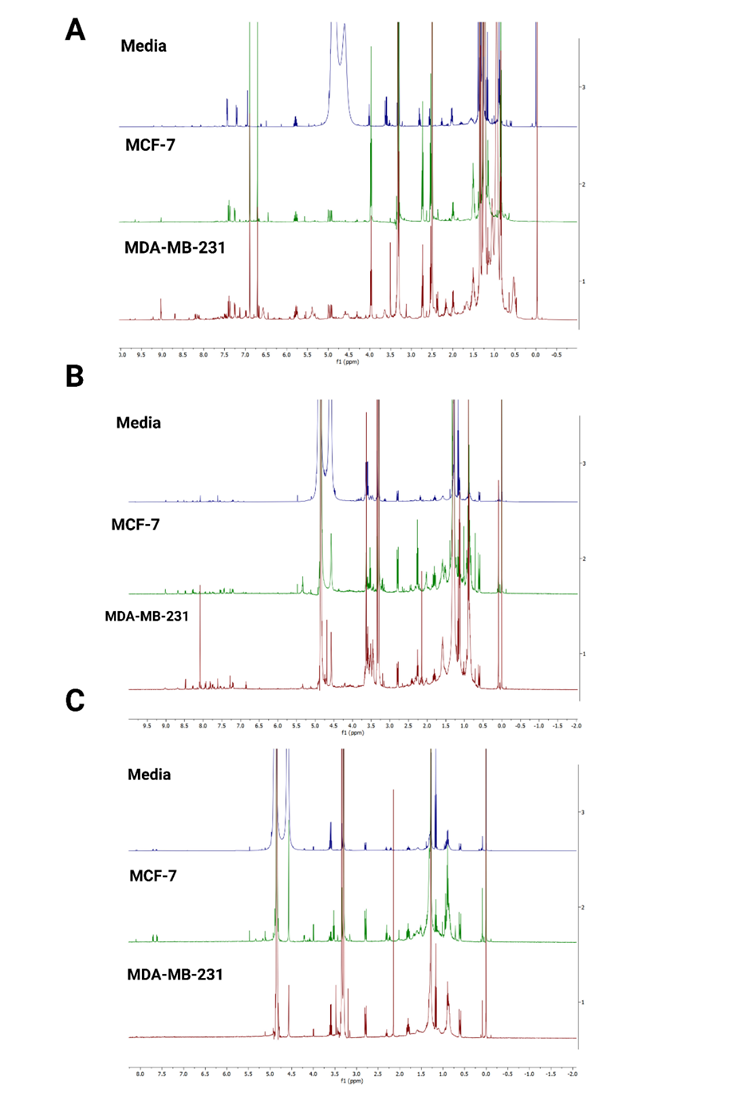


**Figure S1.** Full 500 MHz ^1^H-NMR spectra of Media, MCF-7 and MDA-MB-231 conditioned media extracted using (**A**) ethyl acetate, (**B**) DCM and (**C**) hexane.


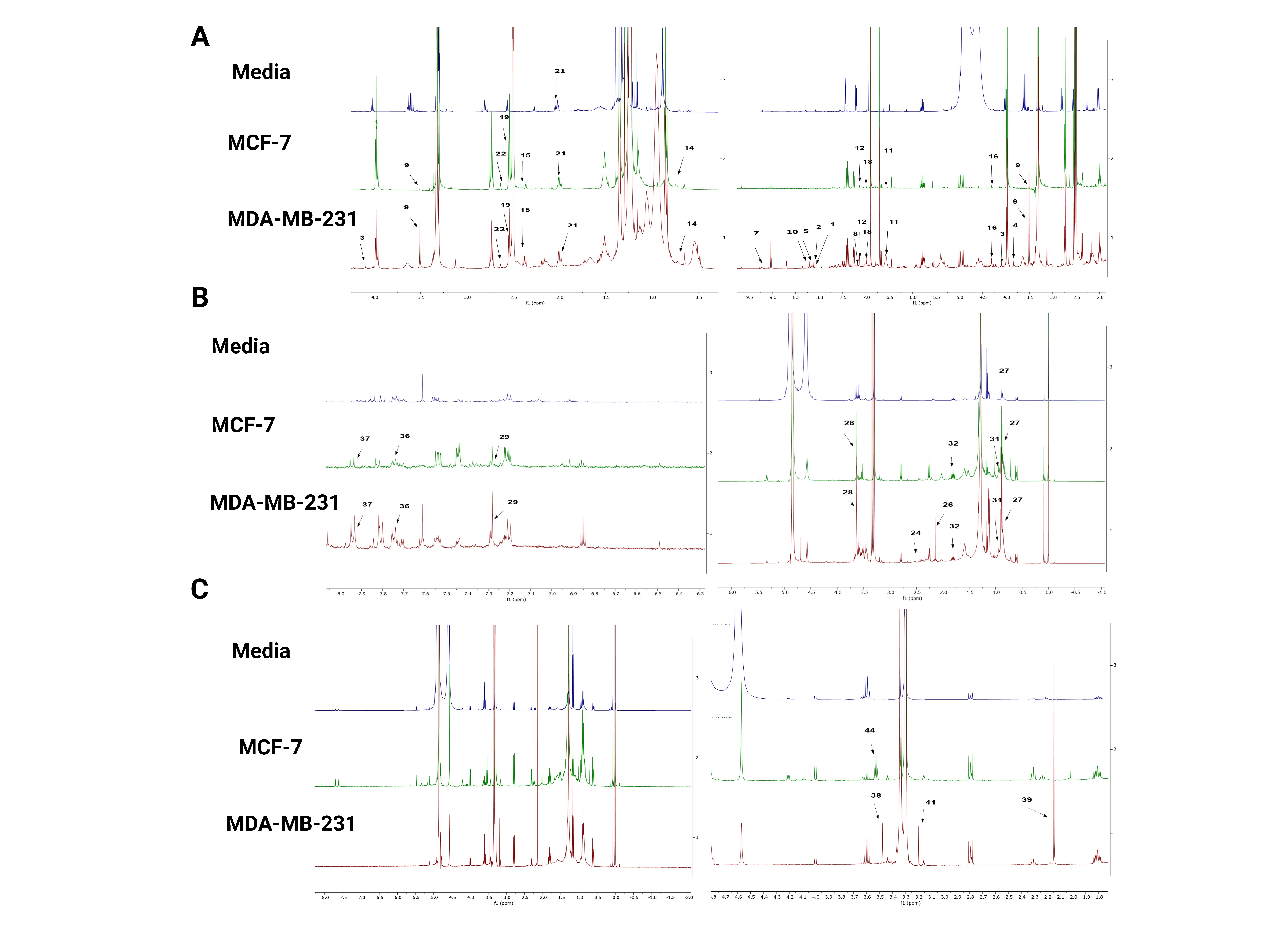


**Figure S2.** Representative 500 MHz ^1^H-NMR spectra of Media, MCF-7 and MDA-MB-231 cells supernatants extracted using (**A**) ethyl acetate, (**B**) DCM and (**C**) hexane showing selected assignment of some of the major metabolic signals.


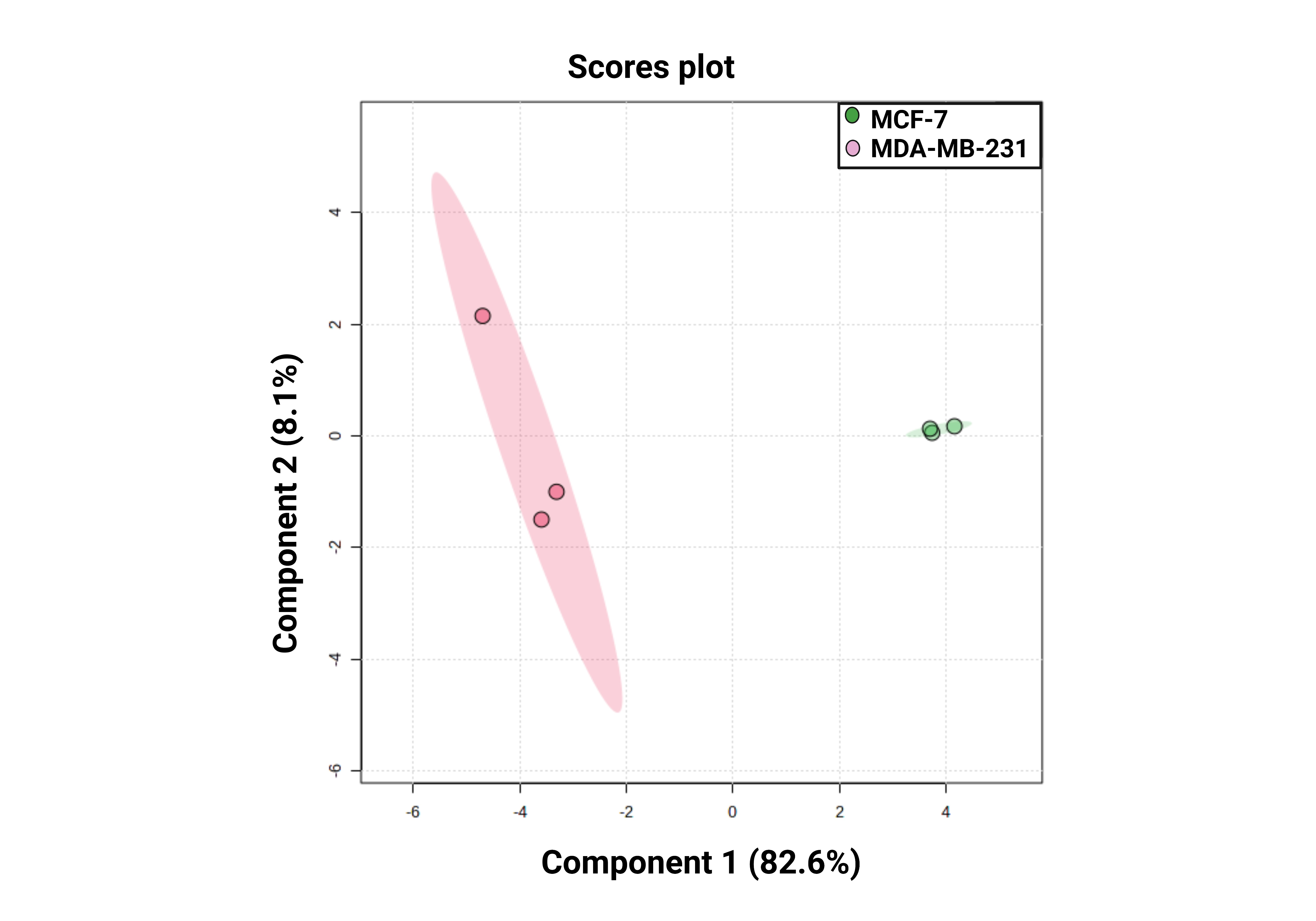


**Figure S3.** PLS-DA of the metabolite profiling of MCF-7 and MDA-MB-231 cell supernatants extracted using ethyl acetate. The red color indicates MDA-MB-231, and green indicates MCF-7 cell supernatants.


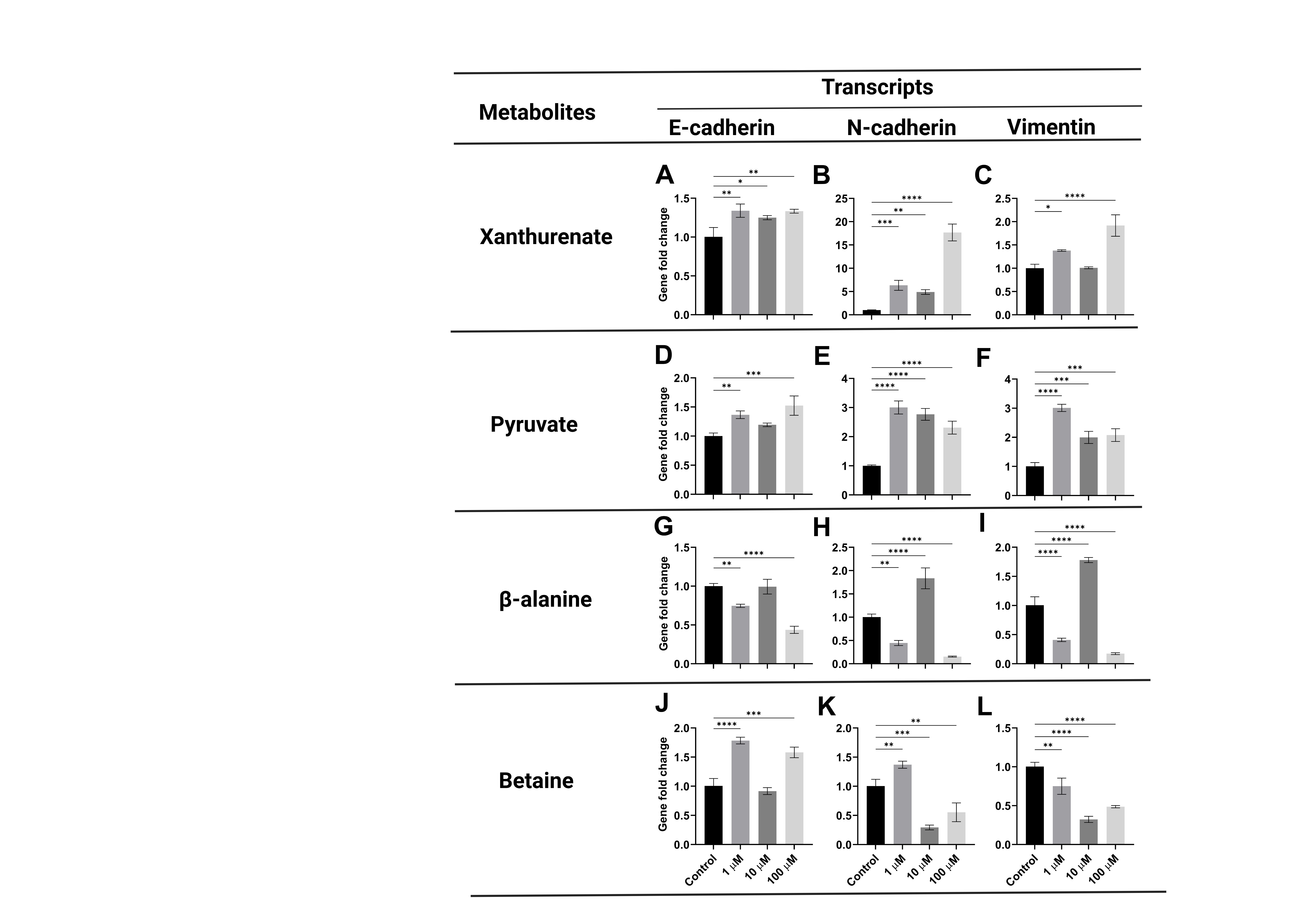


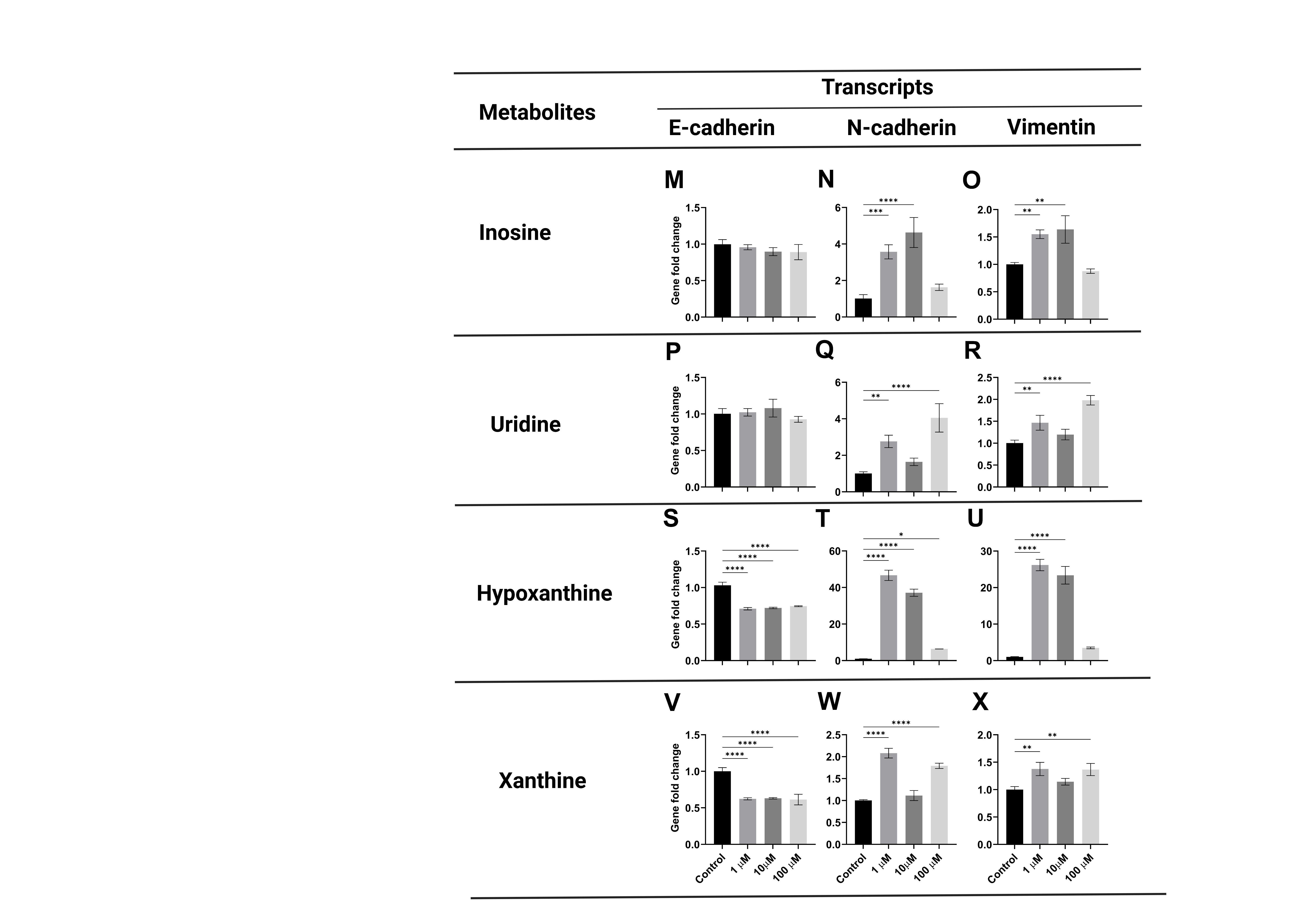


**Figure S4.** Gene expression analysis of E-cadherin, N-cadherin, and vimentin in MCF-7 cells treated with different concentrations of metabolites. (**A-C**) Xanthurenate, (**D-F**) Pyruvate, (**G-I**) β-alanine, (**J-L**) Betaine, (**M-O**) Inosine, (**P-R**) Uridine, (**S-U**) Hypoxanthine and (**V-X**) Xanthine. The data were analysed using one-way ANOVA and Tukey’s multiple comparison test. *P-*value ≤ 0.05 was considered significant. * Reveals that *P*-value < 0.05, ** reveals that *P*-value < 0.01, *** reveals that *P*-value < 0.001, **** reveals that *P*-value < 0.0001.

**
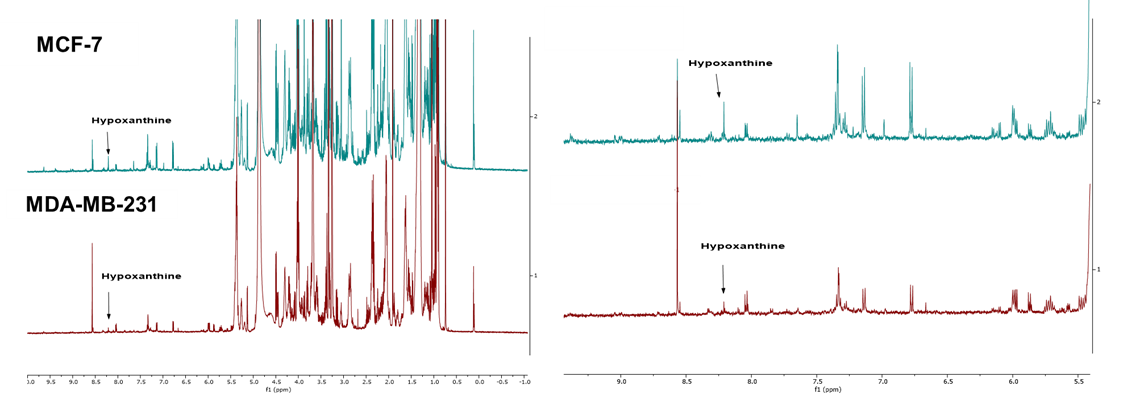
**

**Figure S5. Representative 500 MHz 1H-NMR spectra of the intracellular metabolites of MCF-7 and MDA-MB-231 cells extracted using methanol showing hypoxanthine peak.**


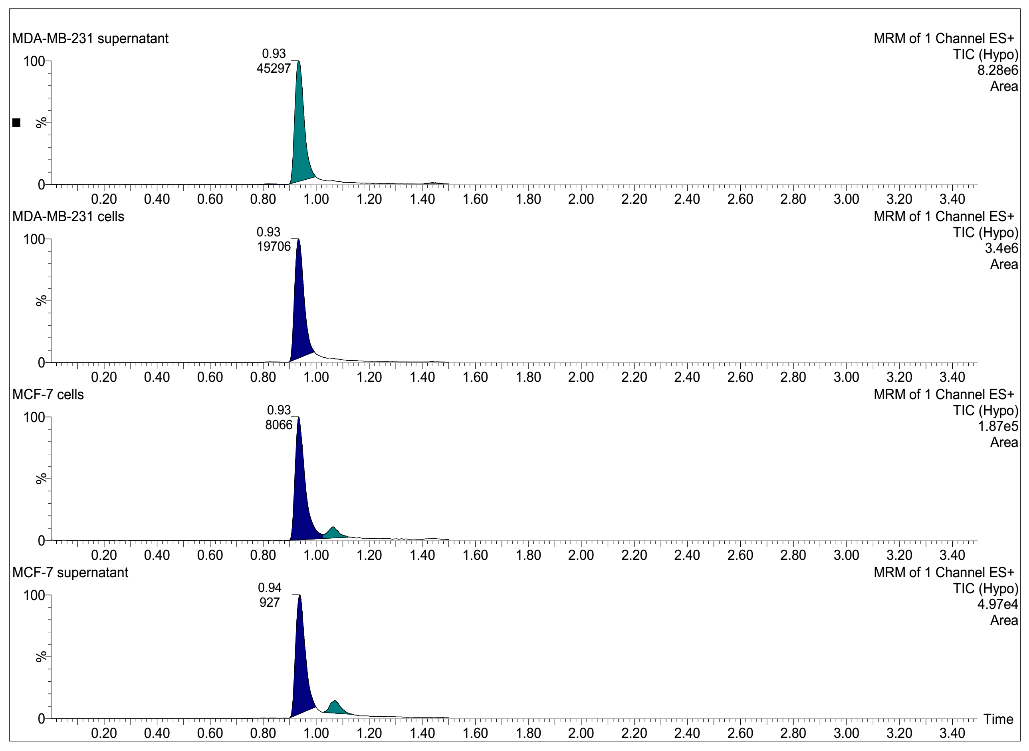


**A**


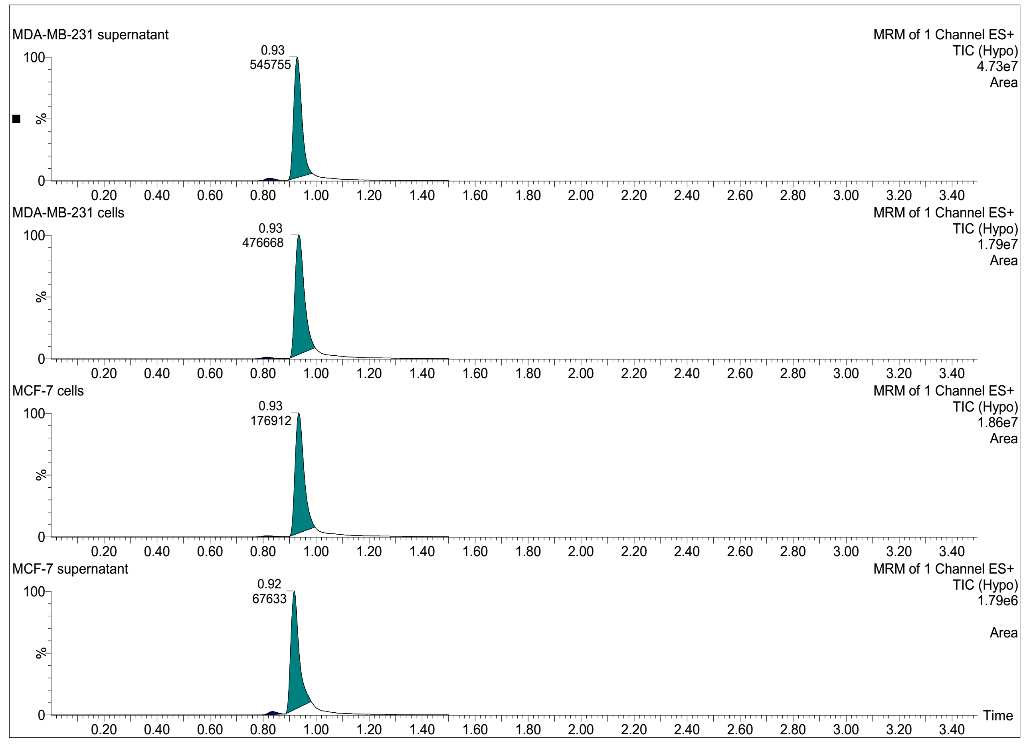


**B**

**Figure S6**. LC chromatograms of (**A**) MDA-MB-231, and MCF-7 cells and supernatant before and (**B**) after spiking at 2µM hypoxanthine.


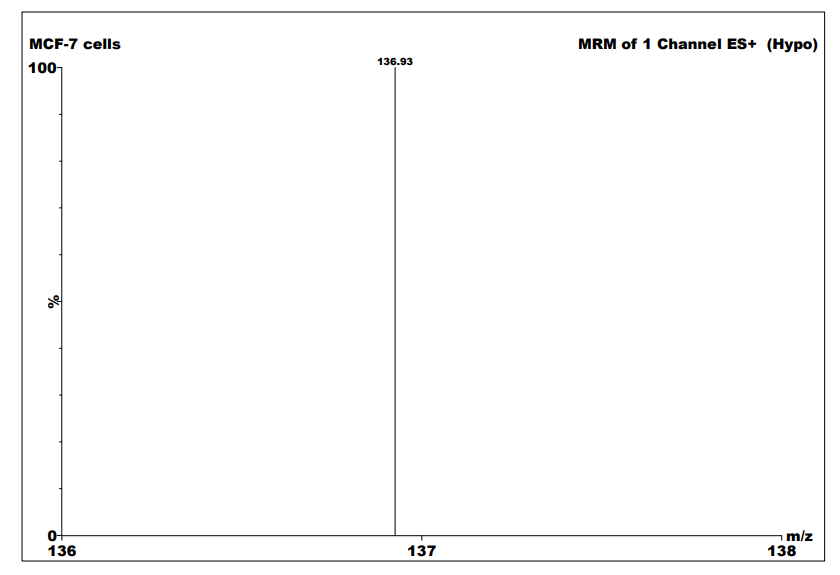


**B**

**A**


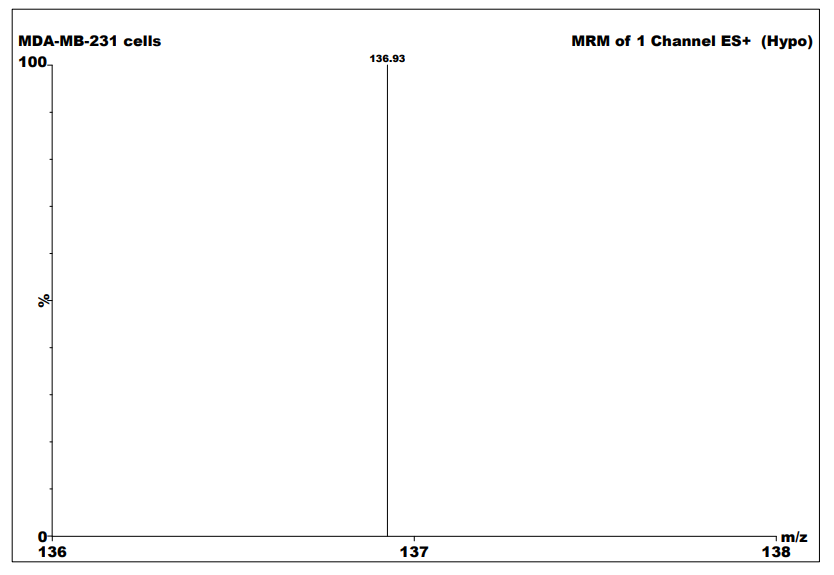


**Figure S7**. LC-MS analysis showing the parent ion peak for HYP in (**A**) MCF-7 and (**B**) MDA-MB-231 cells.

**Figure S8**. Mass spectra of hypoxanthine in (**A**) MDA-MB-231 cells and (**B**) MCF-7 before and after spiking with standard at 2µM hypoxanthine.

**Figure S9.** Original uncropped western blot of Figure 6D. The figure shows the uncropped western blot of PNP and β-actin proteins bands after knockdown of *PNP* gene in comparison to negative control.
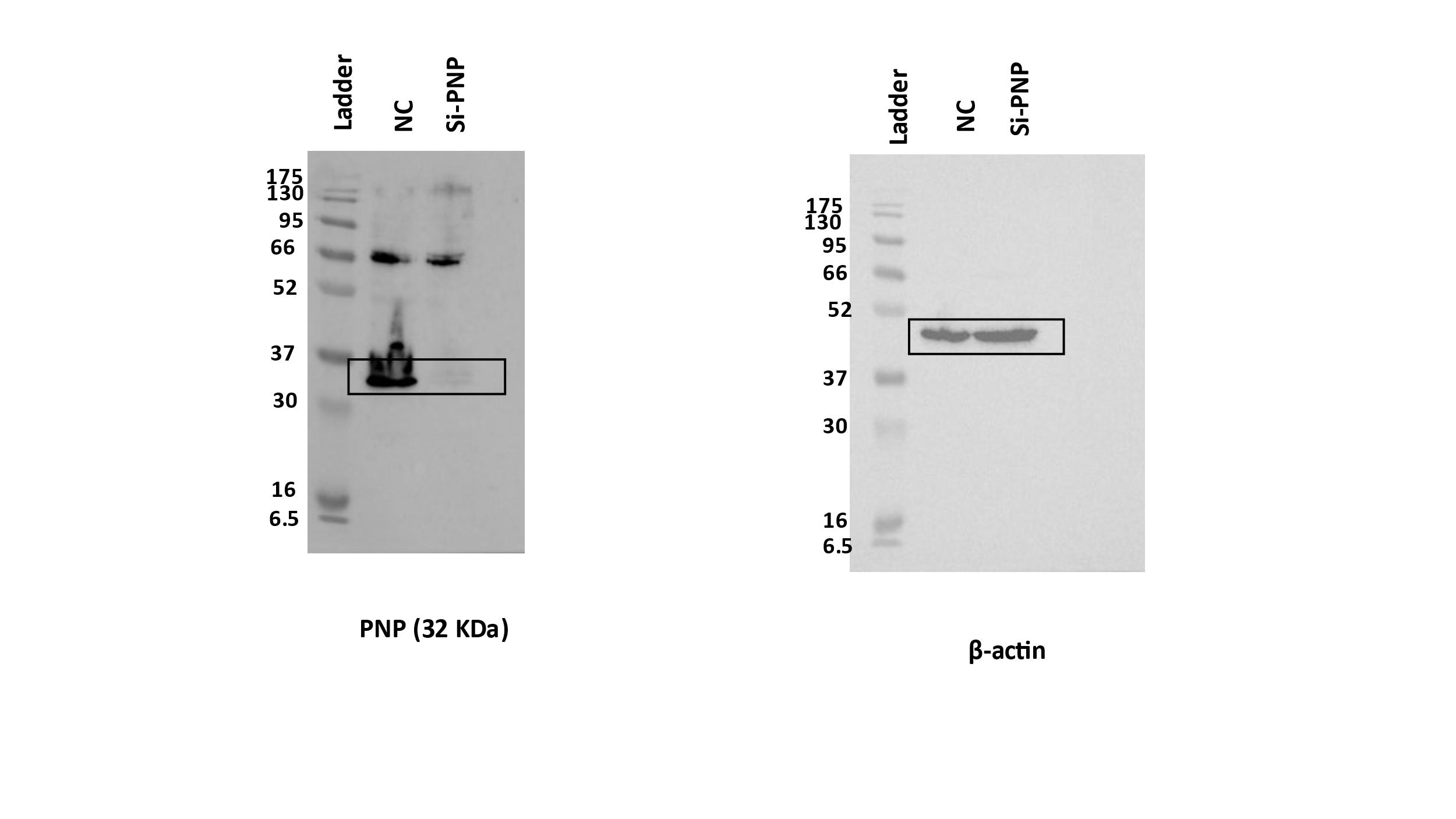


**
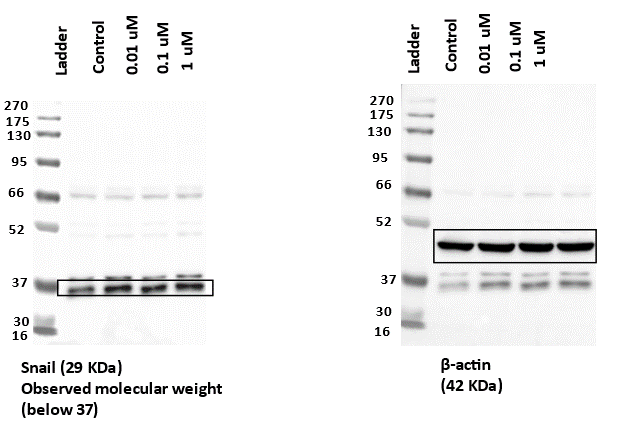
**

**Figure S10.** Original uncropped western blot of Figure 7B. The figure shows the uncropped western blot of Snail and β-actin proteins bands after treatment with hypoxanthine at different concentrations in comparison to negative control.

**
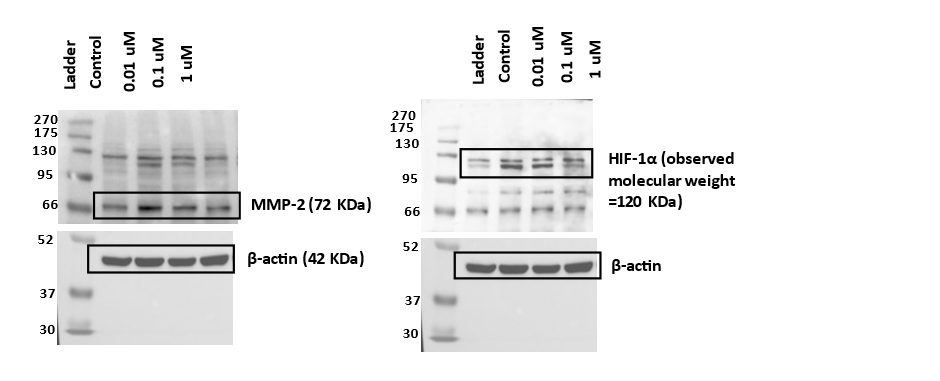
 Figure S11.** Original uncropped western blot of Figures 7D and F. The figure shows the uncropped western blot of (**7D**) MMP-2 and (**7F**) HIF-1α normalized to β-actin proteins bands after treatment with hypoxanthine at different concentrations in comparison to negative control. The membrane was cut at molecular weight 52 KDa. The membrane was probed to HIF-1α and then stripped with mild stripping buffer and probed to MMP-2.

**Supplementary Tables**

**Table S1. Primer sequences used for RT-qPCR experiments.**

| **Target gene** | **Forward primer** | **Reverse primer** | **Reference** |
| --- | --- | --- | --- |
| GAPDH | TGTTGCCATCAATGACCCCTT | CTCCACGACGTACTCAGCG | ^1^ |
| E-cadherin | ATTTTTCCCTCGACACCCGAT | TCCCAGGCGTAGACCAAGA | ^2^ |
| N-cadherin | GCGTCTGTAGAGGCTTCTGG | GCCACTTGCCACTTTTCCTG | ^3^ |
| Vimentin | AGTCCACTGAGTACCGGAGAC | CATTTCACGCATCTGGCGTTC | ^4^ |
| Snail | ACCACTATGCCGCGCTCTT | GGTCGTAGGGCTGCTGGAA | ^5^ |
| HIF-1α | CCTGCACTGAATCAAGAGGTGC | CCATCAGAAGGACTTGCTGGCT | ^6^ |
| PCSK-9 | TGCAAAATCAAGGAGCATGGG | CAGGGAGCACATTGCATCC | ^7^ |
| VEGF | GCAGAATCATCACGAAGTGGTG | CATCAGGGTACTCCTGGAAGAT | ^8^ |
| PDGF | CGACTCCTGGAGATAGAC | GCTTCTCTTCCTCCGAATG | ^9^ |

**Table S2.** **Extracellular metabolites identified by ^1^H-NMR-based profiling of ethyl acetate, DCM and hexane fraction of MDA-MB-231 and MCF-7 cells.** The mean concentration ± standard error is shown for metabolites from a set of three biological replicates.

| **Metabolite** | **ID** | **Chemical shift (ppm)** | **Concentration (mM)*** | | **Fold change** |
| --- | --- | --- | --- | --- | --- |
|  |  |  | **MDA-MB-231 cells** | **MCF-7 cells** |  |
| **Ethyl acetate fraction** |  |  |  |  |  |
| 1-Methylnicotinamide | 1 | 9.27 (s), 8.96 (d), 8.89 (2), 8.17 (t), 4.47 (s) | 1.28 ± 0.05 | 0.00 | - |
| Adenine | 2 | 8.12 (s) | 0.30 ± 0.06 | 0.00 | - |
| Adenosine | 3 | 3.83 (dd), 3.91 (dd), 4.29 (m), 4.42 (dd), 4.79 (dd), 6.05 (d), 8.24 (s), 8.34 (s) | 0.17 ± 0.06 | 0.00 | - |
| Betaine | 4 | 3.25 (s), 3.89 (s) | 0.53 ± 0.13 | 0.00 | - |
| Hypoxanthine | 5 | 8.19 (s), 8.21 (s) | 0.72 ± 0.04 | 0.00 | - |
| Methionine | 6 | 2.16 (m), 2.63 (t), 3.84 (dd) | 1.02 ± 0.51 | 0.00 | - |
| Nicotinurate | 7 | 9.25 (s), 9.00 (m), 8.24 (t), 4.28 (s) | 2.84 ± 1.51 | 0.00 | - |
| Tyramine | 8 | 2.92 (t), 3.23 (t), 6.9 (m), 7.2 (m) | 1.70 ± 0.39 | 0.00 | - |
| Acetoacetate | 9 | 2.28 (s), 3.45 (s) | 6.54 ± 4.04 | 0.21 ± 0.16 | 30.56 |
| Formate | 10 | 8.46 (s) | 0.40 ± 0.09 | 0.12 ± 0.09 | 3.37 |
| Fumarate | 11 | 6.52 (s) | 0.78 ± 0.06 | 0.10 ± 0.08 | 7.53 |
| Histamine | 12 | 7.99 (s), 7.14 (s), 3.29 (t), 3.03 (m) | 0.44 ± 0.08 | 0.21 ± 0.00 | 2.12 |
| Inosine | 13 | 3.84 (dd), 3.92 (dd), 4.27 (m), 4.44 (m), 4.76 (t), 6.11 (d),8.23 (s), 8.35 (s) | 0.19 ± 0.02 | 0.05 ± 0.01 | 3.87 |
| Pantothenate | 14 | 3.97 (s), 3.53 (d), 3.47 (dt), 3.43 (dt), 3.37 (d), 3.32 (d), 2.42 (dt), 0.92 (s), 0.88 (s) | 0.92 ± 0.10 | 0.08 ± 0.03 | 11.03 |
| Pyruvate | 15 | 2.38 (s) | 3.14 ± 0.09 | 0.04 ± 0.01 | 79.74 |
| Uridine | 16 | 3.8 (dd), 3.89 (dd), 4.13 (m), 4.23 (t), 4.36 (t), 5.9 (d),5.92 (d), 7.88 (d) | 0.59 ± 0.02 | 0.06 ± 0.00 | 10.23 |
| Xanthine | 17 | 7.89 (s) | 0.43 ± 0.07 | 0.03 ± 0.00 | 16.53 |
| Xanthurenate | 18 | 7.37 (dd), 7.16 (t), 6.99 (dd), 6.71 (s) | 16.75 ± 1.90 | 0.14 ± 0.11 | 121.34 |
| β-Alanine | 19 | 2.54 (m), 3.17 (m) | 24.98 ± 2.00 | 0.30 ± 0.04 | 83.96 |
| glycerol | 20 | 3.56 (dd), 3.65 (dd), 3.78 (m) | 0.00 | 0.25 ± 0.10 | 0.00 |
| Caprylate | 21 | 2.16 (t), 1.53 (m), 1.27 (d), 0.85 (m) | 6.76 ± 0.27 | 1.26 ± 0.04 | 5.34 |
| 2-Oxobutyrate | 22 | 2.76 (q), 1.06 (t) | 8.08 ± 0.93 | 0.28 ± 0.00 | 28.81 |
| **DCM fraction** |  |  |  |  |  |
| 2-Hydroxyvalerate | 23 | 4.40 (t), 1.69 (td), 1.46 (m), 0.95 (t) | 0.77 ± 0.40 | 0.00 | - |
| 3-Hydroxy-3-methylglutarate | 24 | 1.32 (s), 2.39(d), 2.45(d) | 1.67 ± 0.89 | 0.00 | - |
| Acetoin | 25 | 2.21 (s) | 0.68 ± 0.13 | 0.00 | - |
| Acetone | 26 | 2.24 (s) | 1.46 ± 0.82 | 0.00 | - |
| Caprate | 27 | 2.32 (t), 1.45 (tt), 1.3 (m), 1.28 (m), 1.26 (m), 0.9 (t) | 4.67 ± 0.15 | 0.61 ± 0.05 | 7.60 |
| Glycine | 28 | 3.58 (s) | 11.37 ± 0.27 | 3.84 ± 0.12 | 2.96 |
| Imidazole | 29 | 7.26 (s) | 1.04 ± 0.04 | 0.50 ± 0.00 | 2.07 |
| Isobutyrate | 30 | 2.59 (m), 1.21 (d) | 0.70 ± 0.10 | 0.71 ± 0.01 | 1.00 |
| Isoleucine | 31 | 0.94 (t), 1.01(d) | 0.27 ± 0.01 | 0.21 ± 0.01 | 1.31 |
| Leucine | 32 | 0.96(t), 1.70(m) | 0.70 ± 0.26 | 0.12 ± 0.01 | 5.90 |
| Niacinamide | 33 | 7.6 (dd), 8.26 (m), 8.71 (m), 8.94 (d) | 1.96 ± 0.04 | 0.00 | - |
| Proline | 34 | 4.12 (dd), 3.41 (dt), 3.32 (dt), 2.34 (m), 2.08 (m), 2.00 (m) | 1.35 ± 0.02 | 0.00 | - |
| Succinate | 35 | 2.39 (s) | 0.61 ± 0.20 | 1.40 ± 0.04 | 0.43 |
| Tryptophan | 36 | 3.3 (dd), 3.48 (dd), 4.05 (dd), 7.2 (dd), 7.29 (dd), 7.32 (s),7.54 (d), 7.74 (d), | 0.47 ± 0.01 | 0.00 | - |
| γ-Glutamylphenylalanine | 37 | 1.97 (m),2.34 (m),2.89 (dd),3.21 (dd),3.57 (t),4.46 (m),7.27 (t),7.35 (t),7.91 (d) | 0.61 ± 0.09 | 0.31 ± 0.01 | 1.94 |
| **Hexane Fraction** |  |  |  |  |  |
| 1,3-Dimethylurate | 38 | 3.43 (s), 3.30 (s) | 392.27± 0.40 | 0.00 | - |
| Acetoacetate | 39 | 2.28 (s), 3.45 (s) | 4.75± 0.89 | 0.00 | - |
| Betaine | 40 | 3.25 (s), 3.89 (s) | 6.03± 0.13 | 0.00 | - |
| Malonate | 41 | 3.11 (s) | 4.40± 0.82 | 0.00 | - |
| Valine | 42 | 3.6 (d), 2.26 (m), 1.00 (dd), 0.99 (m), 0.98 (td) | 4.23± 0.47 | 0.00 | - |
| Acetone | 43 | 2.22 (s) | 5.87± 0.27 | 0.1566± 0.12 | 2.96 |
| 4-hydroxybutyrate | 44 | 3.56 (t), 2.58 (t), 1.72 (tt) | 0 | 5.0343± 0.00 | - |

*Note: The mentioned concentration is released from about 2 × 10^7^ cells (about 1 mg of the extract).

**Table S3.** **Literature review about the metabolites extracted using ethyl acetate from MCF-7 or MDA-MB-231 cells illustrating their role in normal and cancer cells and specifically in breast cancer.**

| **Metabolite** | **Identity** | **Role in normal and cancer cells** | **Studies in breast cancer** |
| --- | --- | --- | --- |
| Adenosine | It is a nucleoside composed of adenine and D-ribose ^10^. It is one of the four nucleosides building blocks of RNA ^10^. | It  is an immunosuppressive metabolite produced by cancer cells to stimulate growth, invasion, metastasis, and immune evasion ^11^. | Adenosine promotes proliferation and migration in triple negative breast cancer cells ^12^. |
| Adenine | It is one of the four nucleobases in the nucleic acid of DNA ^13^. | Adenine suppresses colon cancer cells growth through AMP-activated protein kinase mediated autophagy ^14^. Adenine nucleotides stimulate migration in wounded cultures of kidney epithelial cells ^15^. | NA |
| Uridine | It is a pyrimidine nucleoside and one of the five standard nucleosides which make up nucleic acids ^16^. | Uridine di phosphate stimulates intestinal epithelial migration through activation of P2Y6 receptor ^17^. | NA |
| Betaine | It is a small N-trimethylated amino acid ^16^. | It mediates apoptosis and inflammation in prostate cancer cells proliferation by inducing oxidative stress ^18^. In addition, betaine inhibits angiogenesis via inhibiting NF-κB and Akt signaling pathways ^19^. | NA |
| Hypoxanthine and  Xanthine | Hypoxanthine is a purine nucleobase. Xanthine is a product of hypoxanthine degradation by xanthine oxidoreductase (XOR) ^16^. | high levels of HYP was recently identified as a biomarker for human melanoma metastasis ^20^, lung squamous cell carcinoma metastasis ^21^ and prostate cancer aggressiveness ^22^. | Metabolomic analysis of breast cancer tissues showed a significant difference in xanthine between estrogen receptor positive and estrogen receptor negative tissues ^23^. |
| Xanthurenate | Xanthurenate is a product of tryptophan metabolism ^24^. | It induces apoptosis in vascular smooth muscle and retinal pigment epithelium cells ^25^. | It promotes migration of triple negative breast cancer cells via aryl hydrocarbon receptor amplification loop ^26^. |
| Histamine | It is an organic nitrogenous compound involved in local immune responses ^27^. | Histamine exerts its effects through binding to histamine receptors: H1HR, H2HR, H3HR, and H4HR ^27^. In hepatocellular carcinoma, upregulation of H1HR is associated with proliferation and metastasis. H2HR, H3HR, and H4HR enhance proliferation of colon cancer ^28^, human melanoma ^29^, and glioblastoma cells ^30^. | In breast cancer cells, histamine H4 receptor agonists stimulate EMT and promote mammosphere formation through Src and TGF-β signaling ^31^. H3R is suggested to be involved in the regulation of growth and progression of breast cancer cells ^32^. |
| Fumarate | It is an intermediate in the citric acid cycle. It is formed by the oxidation of succinate by the enzyme succinate dehydrogenase ^16^. | It induces EMT through epigenetic modification ^33^. | NA |
| Glycerol | Glycerol links glycolysis and lipid metabolism by reducing dihydroxyacetone phosphate into glycerol-3-phosphate which is used for lipid synthesis ^34^. | Glycerol inhibits cancer cells proliferation ^35^. Besides, it inhibits invasion of the oral cancer cell lines in dose dependent manner ^36^. | Cell proliferation, migration, and invasion of MDA-MB-231 breast cancer cells are inhibited by downregulation of aquaporin 3, which facilitates glycerol transport. ^37^. Glycerol inhibits the proliferation of MCF-7 cells in dose dependent manner ^35^. |
| 1-Methylnicotinamide | It is an endogenous metabolite of nicotinamide ^38^. | In human ovarian cancer, 1-methylnicotinamide plays an immune-regulatory role ^39^. | It prevents breast cancer metastasis ^38^. |
| β-Alanine | β-alanine is a non-essential amino acid ^16^. | Through buffering the formation of lactate from pyruvate, β-alanine supplementation delays lactate accumulation during exercise ^40^. | β-alanine reduces extra-cellular acidity, a factor that promotes tumor invasion ^41^. It has been shown that β-alanine can reduce both proliferation and migration of breast cancer cells without causing cytotoxicity ^41^. |
| Pantothenic acid | Pantothenic acid (vitamin B5) is a water-soluble B vitamin. It is required for the synthesis of coenzyme A (CoA) ^16^. | Pantothenic acid along with β-alanine and glycerophosphoglycerol are associated with a high level of lactate release during glycolysis ^42^. lactate released to the extracellular space of cancer cells was also found to promote cell migration through acidification, which was associated with the metastatic potential of tumors ^42^. | Pantothenic acid is related to increased migration of MDA-MB-231, MDA-MB-435 and JIMT-1 cells due to increased lactic acid release ^42^. |

**References**

S4

1 Ng, J. *et al.* Extracellular matrix components and culture regimen selectively regulate cartilage formation by self-assembling human mesenchymal stem cells in vitro and in vivo. *Stem Cell Res. Ther.* **7**, 1-12, doi:<https://doi.org/10.1186/s13287-016-0447-4> (2016).

2 Yang, X. *et al.* Wnt signaling through Snail1 and Zeb1 regulates bone metastasis in lung cancer. *Am. J. Cancer Res.* **5**, 748 (2015).

3 Sun, H. *et al.* Overexpression of N-cadherin and β-catenin correlates with poor prognosis in patients with nasopharyngeal carcinoma. *Oncol. Lett.* **13**, 1725-1730, doi:<https://doi.org/10.3892/ol.2017.5645> (2017).

4 Jin, Y., Lu, X., Wang, M., Zhao, X. & Xue, L. X-linked inhibitor of apoptosis protein accelerates migration by inducing epithelial–mesenchymal transition through TGF-β signaling pathway in esophageal cancer cells. *Cell Biosci.* **9**, 76, doi:<https://doi.org/10.1186/s13578-019-0338-3> (2019).

5 Feng, H., Lu, J.-J., Wang, Y., Pei, L. & Chen, X. Osthole inhibited TGF β-induced epithelial–mesenchymal transition (EMT) by suppressing NF-κB mediated Snail activation in lung cancer A549 cells. *Cell Adh. Migr.* **11**, 464-475, doi:<https://doi.org/10.1080/19336918.2016.1259058> (2017).

6 Meng, X. *et al.* Hypoxia-inducible factor-1α is a critical transcription factor for IL-10-producing B cells in autoimmune disease. *Nat. Commun.* **9**, 251, doi:<https://doi.org/10.1038/s41467-017-02683-x> (2018).

7 Lebeau, P. F. *et al.* Caffeine blocks SREBP2-induced hepatic PCSK9 expression to enhance LDLR-mediated cholesterol clearance. *Nat. Commun.* **13**, 770, doi:<https://doi.org/10.1038/s41467-022-28240-9> (2022).

8 Hanlon, M. M. *et al.* STAT3 mediates the differential effects of oncostatin M and TNFα on RA synovial fibroblast and endothelial cell function. *Front. Immunol.* **10**, 2056, doi:<https://doi.org/10.3389/fimmu.2019.02056> (2019).

9 Altaie, A. M. *et al.* The essential role of 17-octadecynoic acid in the pathogenesis of periapical abscesses. *J. Endod.*, doi:<https://doi.org/10.1016/j.joen.2022.12.002> (2022).

10 Morelli, M., Simola, N., Popoli, P. & Carta, A. R. in *Handbook of Behavioral Neuroscience* Vol. 20 (eds Heinz Steiner & Kuei Y. Tseng) 201-217 (Elsevier, 2010).

11 Arab, S. & Hadjati, J. Adenosine blockage in tumor microenvironment and improvement of cancer immunotherapy. *Immune Netw.* **19**, doi:<https://doi.org/10.4110/in.2019.19.e23> (2019).

12 Fernandez-Gallardo, M., González-Ramírez, R., Sandoval, A., Felix, R. & Monjaraz, E. Adenosine stimulate proliferation and migration in triple negative breast cancer cells. *PLoS One* **11**, e0167445, doi:<https://doi.org/10.1371/journal.pone.0167445> (2016).

13 Han, M., Cheng, X., Gao, Z., Zhao, R. & Zhang, S. Inhibition of tumor cell growth by adenine is mediated by apoptosis induction and cell cycle S phase arrest. *Oncotarget* **8**, 94286, doi:<https://doi.org/10.18632%2Foncotarget.21690> (2017).

14 Lai, H.-W., Wei, J. C.-C., Hung, H.-C. & Lin, C.-C. Adenine Inhibits the Growth of Colon Cancer Cells via AMP-Activated Protein Kinase Mediated Autophagy. *Evid. Based Complementary Altern. Med.* **2019**, 9151070, doi:<https://doi.org/10.1155/2019/9151070> (2019).

15 Kartha, S. & Toback, F. G. Adenine nucleotides stimulate migration in wounded cultures of kidney epithelial cells. *J. Clin. Investig.* **90**, 288-292, doi:<https://doi.org/10.1172/jci115851> (1992).

16 Wishart, D. S. *et al.* HMDB 5.0: the Human Metabolome Database for 2022. *Nucleic Acids Res.* **50**, D622-d631, doi:<https://doi.org/10.1093/nar/gkab1062> (2022).

17 Nakamura, T., Murata, T., Hori, M. & Ozaki, H. UDP induces intestinal epithelial migration via the P2Y6 receptor. *Br. J. Pharmacol.* **170**, 883-892, doi:<https://doi.org/10.1111/bph.12334> (2013).

18 Kar, F., Hacioglu, C., Kacar, S., Sahinturk, V. & Kanbak, G. Betaine suppresses cell proliferation by increasing oxidative stress–mediated apoptosis and inflammation in DU-145 human prostate cancer cell line. *Cell Stress Chaperones* **24**, 871-881, doi:<https://doi.org/10.1007/s12192-019-01022-x> (2019).

19 Yi, E.-Y. & Kim, Y.-J. Betaine inhibits in vitro and in vivo angiogenesis through suppression of the NF-κB and Akt signaling pathways. *Int. J. Oncol.* **41**, 1879-1885, doi:<https://doi.org/10.3892/ijo.2012.1616> (2012).

20 Kosmopoulou, M. *et al.* Human melanoma-cell metabolic profiling: identification of novel biomarkers indicating metastasis. *Int. J. Mol. Sci.* **21**, 2436, doi:<https://doi.org/10.3390/ijms21072436> (2020).

21 Lee, H. *et al.* Integrative metabolomic and lipidomic profiling of lung squamous cell carcinoma for characterization of metabolites and intact lipid species related to the metastatic potential. *Cancers* **13**, 4179, doi:<https://doi.org/10.3390/cancers13164179> (2021).

22 Dudka, I. *et al.* Comprehensive metabolomics analysis of prostate cancer tissue in relation to tumor aggressiveness and TMPRSS2-ERG fusion status. *BMC cancer* **20**, 1-17, doi:<https://doi.org/10.1186/s12885-020-06908-z> (2020).

23 Budczies, J. *et al.* Comparative metabolomics of estrogen receptor positive and estrogen receptor negative breast cancer: alterations in glutamine and beta-alanine metabolism. *J. Proteomics* **94**, 279-288, doi:<https://doi.org/10.1016/j.jprot.2013.10.002> (2013).

24 Vangipurapu, J., Fernandes Silva, L., Kuulasmaa, T., Smith, U. & Laakso, M. Microbiota-Related Metabolites and the Risk of Type 2 Diabetes. *Diabetes Care* **43**, 1319-1325, doi:<https://doi.org/10.2337/dc19-2533> (2020).

25 Malina, H. Z., Richter, C., Mehl, M. & Hess, O. M. Pathological apoptosis by xanthurenic acid, a tryptophan metabolite: activation of cell caspases but not cytoskeleton breakdown. *BMC physiology* **1**, 1-8, doi:<https://doi.org/10.1186%2F1472-6793-1-7> (2001).

26 Novikov, O. *et al.* An aryl hydrocarbon receptor-mediated amplification loop that enforces cell migration in ER−/PR−/Her2− human breast cancer cells. *Mol. Pharmacol.* **90**, 674-688, doi:<https://doi.org/10.1124/mol.116.105361> (2016).

27 Ohtsu, H. & Seike, M. in *Histamine and Histamine Receptors in Health and Disease* (eds Yuichi Hattori & Roland Seifert) 333-345 (Springer International Publishing

2017).

28 Cianchi, F. *et al.* The role of cyclooxygenase-2 in mediating the effects of histamine on cell proliferation and vascular endothelial growth factor production in colorectal cancer. *Clin. Cancer Res.* **11**, 6807-6815, doi:<https://doi.org/10.1158/1078-0432.ccr-05-0675> (2005).

29 Tomita, K., Nakamura, E. & Okabe, S. Histamine regulates growth of malignant melanoma implants via H2 receptors in mice. *Inflammopharmacology* **13**, 281-289, doi:<https://doi.org/10.1163/156856005774423917> (2005).

30 Lin, J.-J. *et al.* Inhibition of histamine receptor 3 suppresses glioblastoma tumor growth, invasion, and epithelial-to-mesenchymal transition. *Oncotarget* **6**, 17107, doi:<https://doi.org/10.18632/oncotarget.3672> (2015).

31 Galarza, T. E., Delgado, M. A. T., Mohamad, N. A., Martín, G. A. & Cricco, G. P. Histamine H4 receptor agonists induce epithelial-mesenchymal transition events and enhance mammosphere formation via Src and TGF-β signaling in breast cancer cells. *Biochem. Pharmacol.* **180**, 114177, doi:<https://doi.org/10.1016/j.bcp.2020.114177> (2020).

32 Medina, V. *et al.* The role of histamine in human mammary carcinogenesis: H3 and H4 receptors as potential therapeutic targets for breast cancer treatment. *Cancer Biol. Ther.* **7**, 28-35, doi:<https://doi.org/10.4161/cbt.7.1.5123> (2008).

33 Sciacovelli, M. *et al.* Fumarate is an epigenetic modifier that elicits epithelial-to-mesenchymal transition. *Nature* **537**, 544-547, doi:<https://doi.org/10.1038/nature19353> (2016).

34 Blötz, C. & Stülke, J. Glycerol metabolism and its implication in virulence in Mycoplasma. *FEMS Microbiol. Rev.* **41**, 640-652, doi:<https://doi.org/10.1093/femsre/fux033> (2017).

35 Wiebe, J. & Dinsdale, C. Inhibition of cell proliferation by glycerol. *Life Sci.* **48**, 1511-1517, doi:<https://doi.org/10.1016/0024-3205(91)90275-G> (1991).

36 Sakurai, S., Okada, Y. & Mataga, I. Inhibitory effects of glycerol on growth and invasion of human oral cancer cell lines. *J. Hard Tissue Biol.* **20**, 37-46, doi:<http://dx.doi.org/10.2485/jhtb.20.37> (2011).

37 Li, Z. *et al.* The proliferation impairment induced by AQP3 deficiency is the result of glycerol uptake and metabolism inhibition in gastric cancer cells. *Tumor Biol.* **37**, 9169-9179, doi:<https://doi.org/10.1007/s13277-015-4753-8> (2016).

38 Blazejczyk, A. *et al.* 1-methylnicotinamide and its structural analog 1, 4-dimethylpyridine for the prevention of cancer metastasis. *J. Exp. Clin. Cancer Res.* **35**, 1-13, doi:<https://doi.org/10.1186/s13046-016-0389-9> (2016).

39 Kilgour, M. K. *et al.* 1-Methylnicotinamide is an immune regulatory metabolite in human ovarian cancer. *Sci. Adv.* **7**, eabe1174, doi:<https://doi.org/10.1126/sciadv.abe1174> (2021).

40 Schnuck, J. K., Sunderland, K. L., Kuennen, M. R. & Vaughan, R. A. Characterization of the metabolic effect of β-alanine on markers of oxidative metabolism and mitochondrial biogenesis in skeletal muscle. *J. Nutr. Biochem.* **20**, 34, doi:<https://doi.org/10.20463%2Fjenb.2016.06.20.2.5> (2016).

41 Vaughan, R. A. *et al.* β-alanine suppresses malignant breast epithelial cell aggressiveness through alterations in metabolism and cellular acidity in vitro. *Mol. Cancer* **13**, 1-10, doi:<https://doi.org/10.1186/1476-4598-13-14> (2014).

42 Hutschenreuther, A., Birkenmeier, G., Bigl, M., Krohn, K. & Birkemeyer, C. Glycerophosphoglycerol, beta-alanine, and pantothenic acid as metabolic companions of glycolytic activity and cell migration in breast cancer cell lines. *Metabolites* **3**, 1084-1101, doi:<https://doi.org/10.3390%2Fmetabo3041084> (2013).
